# IGF1-mediated HOXA13 overexpression promotes colorectal cancer metastasis through upregulating ACLY and IGF1R

**DOI:** 10.1038/s41419-021-03833-2

**Published:** 2021-06-01

**Authors:** Chenyang Qiao, Wenjie Huang, Jie Chen, Weibo Feng, Tongyue Zhang, Yijun Wang, Danfei Liu, Xiaoyu Ji, Meng Xie, Mengyu Sun, Daiming Fan, Kaichun Wu, Limin Xia

**Affiliations:** 1 State key Laboratory of Cancer Biology, National Clinical Research Center for Digestive Diseases and Xijing Hospital of Digestive Diseases, Fourth Military Medical University, 710032 Xi’an, Shaanxi China; 2 Department of Gastroenterology, Institute of Liver and Gastrointestinal Diseases, Hubei Key Laboratory of Hepato-Pancreato-Biliary Diseases, Tongji Hospital of Tongji Medical College, Huazhong University of Science and Technology, 430030 Wuhan, Hubei China; 3 Hubei Key Laboratory of Hepato-Pancreato-Biliary Diseases; Hepatic Surgery Center, Tongji Hospital, Tongji Medical College, Huazhong University of Science and Technology; Clinical Medicine Research Center for Hepatic Surgery of Hubei Province; Key Laboratory of Organ Transplantation, Ministry of Education and Ministry of Public Health, 430030 Wuhan, Hubei China

**Keywords:** Metastasis, Colorectal cancer

## Abstract

Metastasis is the major reason for the high mortality of colorectal cancer (CRC) patients and its molecular mechanism remains unclear. Here, we report a novel role of Homeobox A13 (HOXA13), a member of the Homeobox (HOX) family, in promoting CRC metastasis. The elevated expression of HOXA13 was positively correlated with distant metastasis, higher AJCC stage, and poor prognosis in two independent CRC cohorts. Overexpression of HOXA13 promoted CRC metastasis whereas downregulation of HOXA13 suppressed CRC metastasis. Mechanistically, HOXA13 facilitated CRC metastasis by transactivating ATP-citrate lyase (ACLY) and insulin-like growth factor 1 receptor (IGF1R). Knockdown of ACLY and IGFIR inhibited HOXA13-medicated CRC metastasis, whereas ectopic overexpression of ACLY and IGFIR rescued the decreased CRC metastasis induced by HOXA13 knockdown. Furthermore, Insulin-like growth factor 1 (IGF1), the ligand of IGF1R, upregulated HOXA13 expression through the PI3K/AKT/HIF1α pathway. Knockdown of HOXA13 decreased IGF1-mediated CRC metastasis. In addition, the combined treatment of ACLY inhibitor ETC-1002 and IGF1R inhibitor Linsitinib dramatically suppressed HOXA13-mediated CRC metastasis. In conclusion, HOXA13 is a prognostic biomarker in CRC patients. Targeting the IGF1-HOXA13-IGF1R positive feedback loop may provide a potential therapeutic strategy for the treatment of HOXA13-driven CRC metastasis.

## Introduction

Metastasis is the main reason for the high mortality rate in patients with colorectal cancer (CRC)^1^. Nowadays, CRC ranks as the fourth most deadly cancer worldwide with ~900,000 deaths annually^2^. Although many efforts have been made to improve the survival outcomes, few patients with CRC recurrence and metastasis really receive a long-term benefit^3^. Thus, investigating the sophisticated mechanisms of metastatic CRC is of great significance, and it is urgently needed to develop a new therapeutic strategy to inhibit CRC metastasis.

Homeobox (HOX) genes, which encode an evolutionarily highly conserved transcription factor family, function as key determinants in embryogenesis by regulating cell proliferation, differentiation, apoptosis, receptor signaling, angiogenesis, and metabolism^4^. Mammalians have 39 HOX genes, which are distributed into four gene clusters: HOXA, HOXB, HOXC, and HOXD^4^. HOXA subfamily, one cluster of HOX genes, comprises 11 members and behaves as both oncogenic transcriptional factors and tumor suppressor genes (TSGs) exerting pleiotropic effects in the malignant initiation and progression in varieties of tumors. For example, HOXA1^5^, HOXA2^6^, HOXA7^7^, HOXA9^8^, HOXA10^9^, HOXA11^10^, and HOXA13^11^ expression were elevated in several cancers and functioned as oncogenes to promote cancer invasion and metastasis. In contrast, HOXA4^12^ and HOXA5^13^ expression were downregulated in human cancers and served as TSGs to inhibit cancer invasion and metastasis. Therefore, HOXA subfamily genes are critical regulators in cancer metastasis. Several studies have reported that HOXA13, a member of the HOXA subfamily, was significantly elevated in several cancers, and was associated with TNM stage, lymph nodes metastasis, and unfavorable clinical outcome in ovarian cancer^14^, pancreatic cancer^15^, and bladder cancer^16^. Overexpression of HOXA13 promoted migration and invasion through the induction of epithelial–mesenchymal-transition (EMT) in cancer cell lines^17,18^. A recent study reported that elevated HOXA13 expression was associated with histological grade, T stage, N stage, and tumor size in CRC tissues. In vitro studies showed that HOXA13 promoted colon cancer cell proliferation, migration, and invasion, and in vivo studies showed that HOXA13 promoted tumor formation through the Wnt/β-Catenin pathway^19^. However, the regulatory mechanism of HOXA13 overexpression in CRC remains unknown. The molecular mechanism underlying HOXA13-mediated CRC metastasis needs further investigation.

Insulin-like growth factor 1 receptor (IGF1R) is identified as one cell surface marker that comprises a ligand-binding domain and the tyrosine kinase domain^20^. The interaction of IGF1R by its ligand, insulin-like growth factor 1(IGF1), activates signaling pathways such as phosphatidylinositol-3 kinase (PI3K)/AKT and Ras/Raf/extracellular signal-regulated kinase (ERK)^21^. Mounting evidence has demonstrated that the IGF1-IGF1R signaling promotes initiation and progression of many cancer types^22,23^, including CRC^24,25^. Elevation of IGF1R expression is associated with the progression, development, and poor prognosis in CRC^26,27^. Moreover, high IGF1 level contributes to colorectal carcinogenesis, while inhibiting IGF1 suppresses CRC cell growth and metastasis^28^. These findings indicate that IGF1-IGF1R signaling is critical for CRC progression and metastasis.

In this research, we found that IGF1 upregulated HOXA13 expression through IGF1R/PI3K/AKT/HIF1α signaling pathway. HOXA13 promoted CRC metastasis through transactivating ACLY and IGF1R expression, which formed an IGF1-HOXA13-IGF1R positive feedback loop. The combination treatment of ACLY inhibitor ETC-1002 and IGF1R inhibitor Linsitinib significantly suppressed HOXA13-mediated CRC metastasis.

## Results

### Overexpression of HOXA13 promotes CRC metastasis and indicates a worse prognosis

**Fig. 1:**
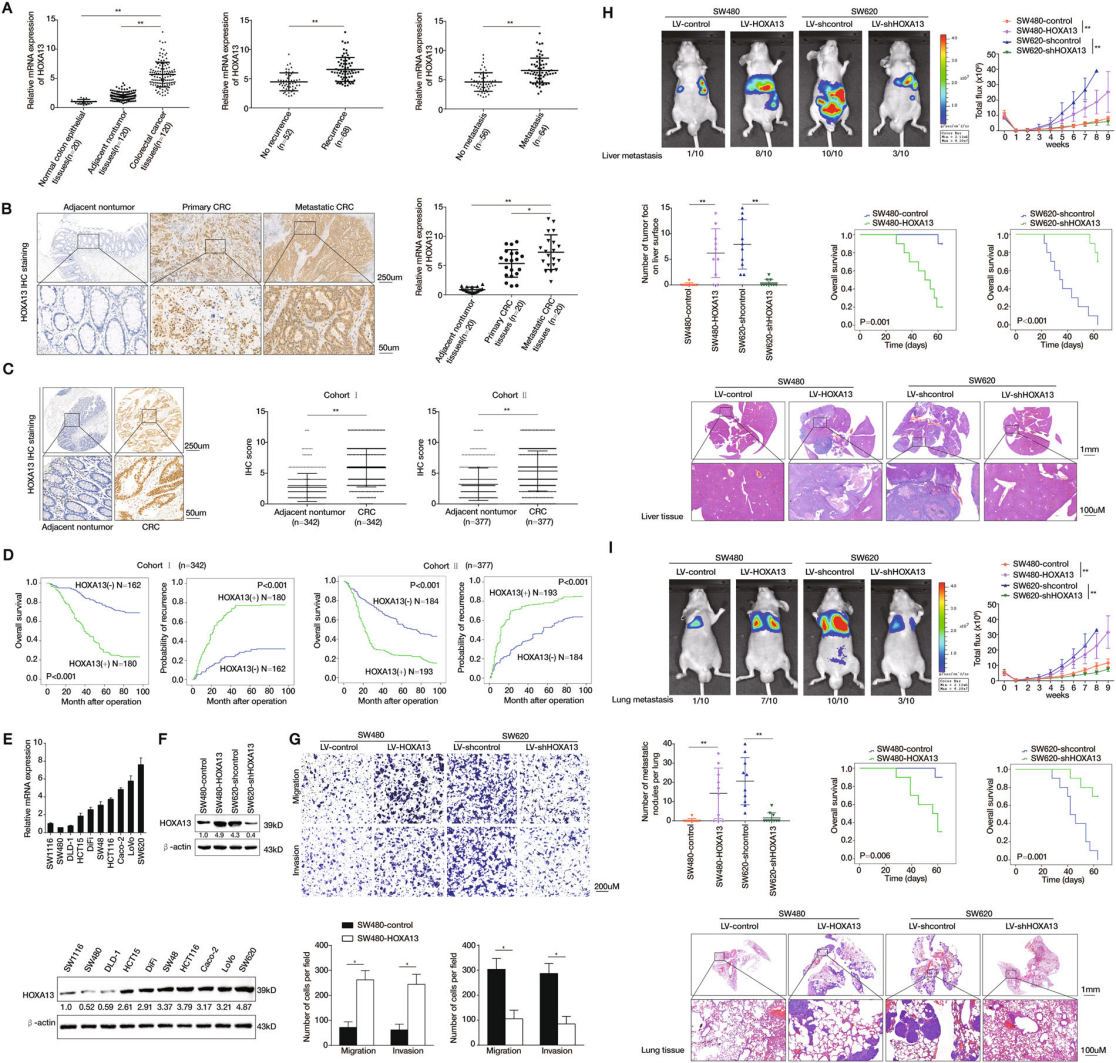
Overexpression of HOXA13 promotes CRC metastasis and indicates worse prognosis. **A** (Left) Real-time PCR analysis of HOXA13 expression in 120 pairs of CRC and adjacent nontumor specimens, and normal colorectal epithelial specimens (*n* = 20). (Middle) The mRNA expression of HOXA13 in CRC specimens from patients with recurrence (*n* = 52) or without recurrence (*n* = 68). (Right) The mRNA expression of HOXA13 in CRC specimens from patients with metastasis (*n* = 56) or without metastasis (*n* = 64). **B** The protein and mRNA expression of HOXA13 were detected by IHC staining (left) and real-time PCR (right) analyses in 20 pairs of normal colorectal epithelial tissues, primary colon cancer, and paired metastatic CRC tissues. The scale bars represent 250 μm (low magnification) and 50 μm (high magnification). **C** (Left)The representative image of IHC staining of HOXA13 in CRC and adjacent nontumor tissues microarray. The scale bars represent 250 μm (low magnification) and 50 μm (high magnification). (Right) The IHC score of HOXA13 in CRC and adjacent nontumor samples in two independent cohorts. **D** Kaplan–Meier analysis of the relationships of HOXA13 expression and overall survival times or the recurrence rates in two independent CRC cohorts. **E** Real-time PCR (upper) and western blotting (lower) analyses of the mRNA and protein expression of HOXA13 in CRC cell lines. **F** Western blotting (lower) analysis of HOXA13 protein expression in SW480 and SW620 cells after lentivirus transfection. **G** Cell migratory and invasive capabilities in the indicated CRC cell lines by Transwell assay. **H** In vivo liver metastatic assays. Four stable cell lines were injected into the spleens of nude mice (*n* = 10 mice per group). Representative bioluminescent imaging, intensity of bioluminescence signals, number of metastatic liver nodules, overall survival times, liver tissues’ HE staining in each group (*n* = 10), and the incidence of liver metastasis were shown. The scale bars represent 1 mm (low magnification) and 100 μm (high magnification). **I** In vivo lung metastatic assays. Four stable cell lines were injected into the tail veins of nude mice (*n* = 10 mice per group). Representative bioluminescent imaging, intensity of bioluminescence signals, number of metastatic lung nodules, overall survival times, lung tissues’ HE staining in each group and the incidence of lung metastasis were shown. The scale bars represent 1 mm (low magnification) and 100 μm (high magnification). *P < 0.05 ** *P* < 0.01. Data are displayed as the mean ± s.d.

Firstly, we examined the *HOXA13* mRNA expression in a cohort of 120 paired CRC and adjacent nontumor specimens, and 20 normal colorectal epithelial specimens. The mRNA levels of *HOXA13* were markedly upregulated in CRC compared with that in adjacent nontumor tissues and normal colorectal epithelial tissues. CRC patients who experienced metastasis or recurrence displayed significantly higher *HOXA13* mRNA levels than those who did not (Fig. 1A). Besides, HOXA13 expression was detected in samples of 20 paired primary CRC, metastatic CRC, and adjacent noncancerous samples. The mRNA and protein expression of HOXA13 was distinctly elevated in metastatic CRC than samples of primary CRC and adjacent nontumor specimens (Fig. 1B).

Next, the expression and clinical value of HOXA13 were detected in two human CRC cohorts. IHC staining exhibited that HOXA13 expression was higher in CRC than adjacent noncancerous samples (Fig. 1C). The elevated HOXA13 expression was associated with worse tumor differentiation, distant metastasis, and higher AJCC stage (Table 1). CRC patients with positive HOXA13 expression had higher recurrence rates and shorter overall survival than CRC patients with negative expression of HOXA13 (Fig. 1D). Multivariate analysis indicated that HOXA13 expression was an independent and significant risk factor for CRC patients’ recurrence and survival (Table 2).

**Table 1:** Correlation between HOXA13 expression and clinicopathological characteristics of CRC in two independent cohorts of human CRC tissues.

	Cohort I (*n* = 342)			Cohort II (*n* = 377)		
Clinicopathological variables	Tumor HOXA13 expression		*P*-value	Tumor HOXA13 expression		*P-*value
	Negative (*n* = 162)	Positive (*n* = 180)		Negative (*n* = 184)	Positive (*n* = 193)	
Age	67.22 (10.30)	65.31 (12.09)	0.120	67.44 (11.81)	67.56 (11.42)	0.921
Sex						
Female	63	87	0.079	77	92	0.300
Male	99	93		107	101	
Tumor location						
Right colon	80	70	0.133	68	92	0.074
Left colon	65	84		91	74	
Rectum	17	26		25	27	
Tumor size						
<5 cm	70	74	0.742	66	77	0.458
≥5 cm	92	106		118	116	
Tumor differentiation						
Well or moderate	154	79	<0.001	125	87	<0.001
Poor	8	101		59	106	
Tumor invasion						
T1	6	1	<0.001	10	5	0.002
T2	24	6		5	19	
T3	111	113		139	122	
T4	21	60		30	47	
Lymph node metastasis						
Absent	153	32	<0.001	161	52	<0.001
Present	9	148		23	141	
Distant metastasis						
Absent	160	117	<0.001	172	133	<0.001
Present	2	63		12	60	
AJCC stage						
Stage I	30	5	<0.001	12	7	<0.001
Stage II	125	20		149	8	
Stage III	5	92		11	90	
Stage IV	2	63		12	58

**Table 2:** Univariate and multivariate analysis of factors associated with survival and recurrence in two independent cohorts of human CRC.

Clinical variables	Time to recurrence		Overall survival	
	HR (95% CI)	*P*-value	HR (95% CI)	*P*-value
Cohort I (*n* = 342)				
Univariate analysis				
Age (≤50 versus > 50)	0.998 (0.985–1.011)	0.764	0.998 (0.985–1.011)	0.740
Sex (female versus male)	1.212 (0.913–1.610)	0.183	1.161 (0.872–1.547)	0.306
Tumor size (≤5 versus >5 cm)	0.840 (0.629–1.121)	0.237	0.824 (0.615–1.105)	0.196
Tumor differentiation (well/moderate versus poor)	0.184 (0.137–0.247)	<0.001	0.185 (0.138–0.249)	<0.001
Tumor invasion (T1–T3 versus T4)	0.348 (0.258–0.469)	<0.001	0.354 (0.262–0.479)	<0.001
Lymph node metastasis (absent versus present)	0.144 (0.103–0.200)	<0.001	0.141 (0.101–0.197)	<0.001
Distant metastasis (absent versus present)	0.112 (0.080–0.158)	0.001	0.113 (0.081–0.158)	0.237
AJCC stage (I–II versus III–IV)	0.140 (0.100–0.196)	<0.001	0.138 (0.098–0.194)	<0.001
HOXA13 expression (negative versus positive)	0.254 (0.184–0.350)	<0.001	0.247 (0.178–0.343)	<0.001
Multivariate analysis				
Tumor differentiation (well/moderate versus poor)	0.722 (0.485–1.076)	0.109	0.746 (0.498–1.118)	0.156
Tumor invasion (T1–T3 versus T4)	0.564 (0.399–0.796)	0.001	0.600 (0.422–0.852)	0.004
Lymph node metastasis (absent versus present)	0.396 (0.142–1.102)	0.076	0.369 (0.129–1.056)	0.063
Distant metastasis (absent versus present)	0.350 (0.233–0.528)	<0.001	0.341 (0.226–0.515)	<0.001
AJCC stage (I–II versus III–IV)	0.260 (0.080–0.848)	0.026	0.274 (0.082–0.922)	0.036
HOXA13 expression (negative versus positive)	2.443 (1.339–4.460)	0.004	2.442 (1.319–4.521)	0.004
Cohort II (*n* = 377)				
Univariate analysis				
Age (≤50 versus > 50)	0.997 (0.987–1.008)	0.620	0.999 (0.988–1.010)	0.798
Sex (female versus male)	1.091 (0.862–1.380)	0.467	1.137 (0.895–1.445)	0.294
Tumor size (≤5 versus >5 cm)	0.904 (0.710–1.151)	0.413	0.878 (0.685–1.125)	0.303
Tumor differentiation (well/moderate versus poor)	0.483 (0.381–0.612)	<0.001	0.462 (0.363–0.588)	<0.001
Tumor invasion (T1–T3 versus T4)	0.634 (0.482–0.834)	<0.001	0.635 (0.480–0.840)	0.001
Lymph node metastasis (absent versus present)	0.202 (0.157–0.261)	<0.001	0.179 (0.138–0.233)	<0.001
Distant metastasis (absent versus present)	0.137 (0.100–0.187)	0.011	0.117 (0.085–0.161)	<0.001
AJCC stage (I–II versus III–IV)	0.187 (0.1458–0.242)	<0.001	0.166 (0.127–0.216)	<0.001
HOXA13 expression (negative versus positive)	0.462 (0.364–0.588)	<0.001	0.410 (0.320–0.526)	<0.001
Multivariate analysis				
Tumor differentiation (well/moderate versus poor)	0.823 (0.632–1.071)	0.148	0.823 (0.627–1.079)	0.158
Tumor invasion (T1–T3 versus T4)	0.677 (0.509–0.899)	0.007	0.686 (0.513–0.918)	0.011
Lymph node metastasis (absent versus present)	1.451 (0.675–3.121)	0.341	1.209 (0.562–2.602)	0.627
Distant metastasis (absent versus present)	0.353 (0.249–0.501)	<0.001	0.312 (0.219–0.445)	<0.001
AJCC stage (I–II versus III–IV)	0.110 (0.046–0.262)	<0.001	0.122 (0.051–0.292)	<0.001
HOXA13 expression (negative versus positive)	1.769 (1.225–2.554)	0.002	1.667 (1.138–2.442)	0.009

HOXA13 expression levels were examined in several CRC cell lines. The results indicated that HOXA13 mRNA and protein expression was higher in CRC cells that have the high capabilities to metastasis (LoVo and SW620) than those that have low metastatic capabilities (SW480 and DLD-1) (Fig. 1E). Then, SW480 cells with relatively low HOXA13 expression and SW620 cells with relatively high HOXA13 expression were selected to construct SW480-HOXA13 and SW620-shHOXA13 stable cell lines (Fig. 1F). Upregulation of HOXA13 expression elevated the SW480 cells’ migratory and invasive abilities. Downregulation of HOXA13 expression suppressed the SW620 cells’ migratory and invasive capabilities (Fig. 1G).

The liver metastatic assays indicated that HOXA13 overexpression elevated the intensity of bioluminescent imaging (BLI) signals and the liver metastasis incidence with its metastatic nodules’ number, and lowed overall survival times in the SW480-HOXA13 group. While, downregulation of HOXA13 reduced the intensity of BLI signals and the liver metastasis incidence with its metastatic nodules’ number, and prolonged overall survival times in the SW620-shHOXA13 group (Fig. 1H). Similarly, the lung metastatic assay indicated that HOXA13 overexpression elevated the BLI signals’ intensity and the lung metastasis incidence with its metastatic nodules’ number, and reduced the SW480-HOXA13 group’s overall survival. Nevertheless, downregulation of HOXA13 decreased the BLI signals’ intensity and the lung metastasis incidence with its metastatic nodules’ number, and elevated the SW620-shHOXA13 group’s overall survival (Fig. 1I).

### HOXA13 promotes CRC metastasis via upregulating ACLY and IGF1R

**Fig. 2:**
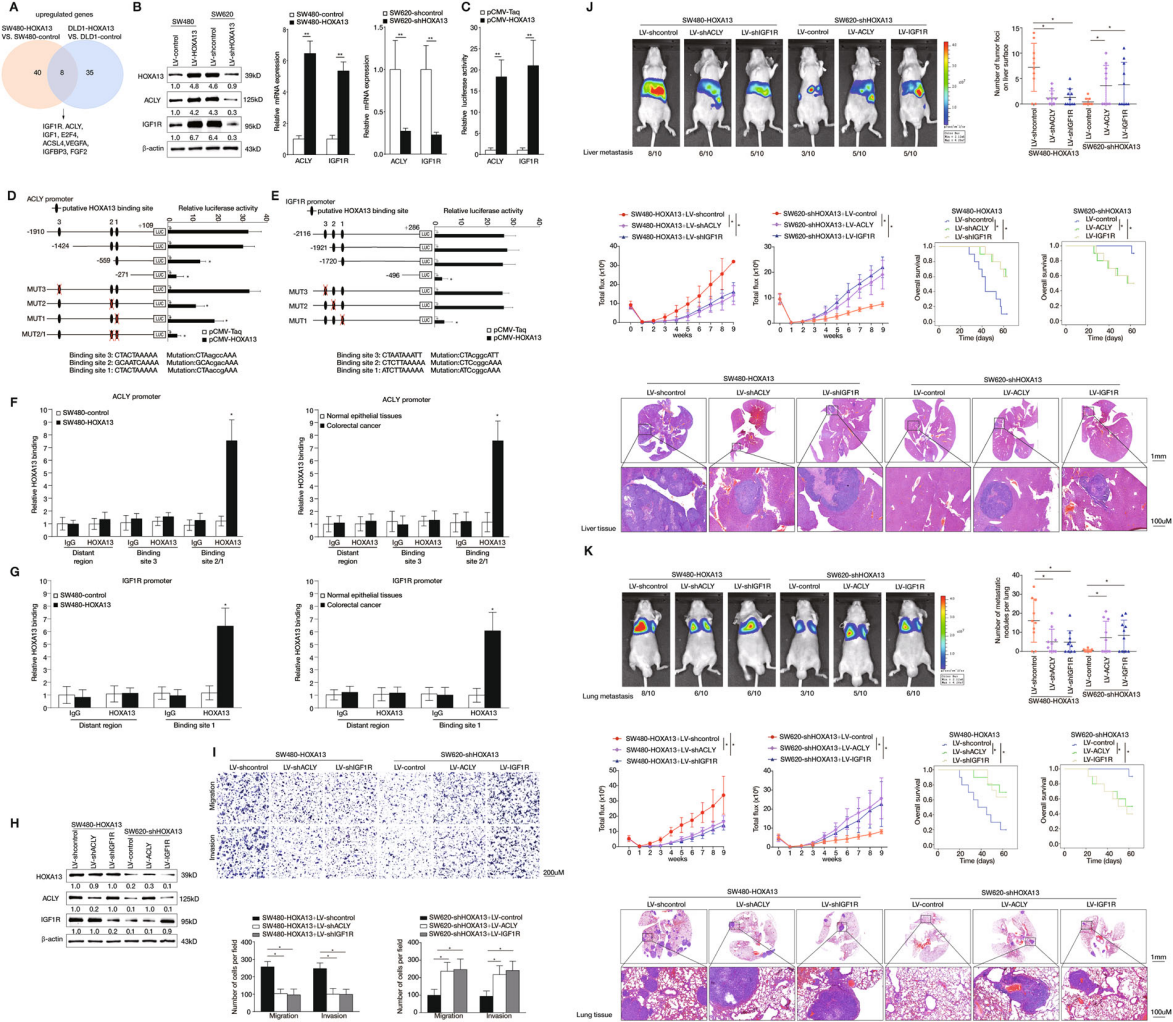
HOXA13 promotes CRC metastasis via upregulating ACLY and IGF1R. **A** Venn diagram presenting the overlap of upregulated genes in both SW480-HOXA13 and DLD-1-HOXA13 cell lines (fold change >2.0). **B** (Left) Western blotting analysis of ACLY and IGF1R expression in the corresponding CRC cell lines after lentivirus transfection. (Right) Real-time PCR analysis of *ACLY* and *IGF1R* expression in the corresponding CRC cell lines after lentivirus transfection. **C** Luciferase reporter assay displayed the promoter activities of *ACLY* and *IGF1R* after SW480 cells were co-transfected with pCMV-HOXA13 and *ACLY* or *IGF1R* promoter constructs. **D**, **E** HOXA13-responsive regions in the *ACLY* and *IGF1R* promoter were analyzed by deletion and selective mutation. Serially truncated and mutated *ACLY* or *IGF1R* promoter constructs were co-transfected with pCMV-HOXA13, and relative luciferase activities were determined. Left-component was the schematic constructs, and right-component presented the relative luciferase activities. **F**, **G** ChIP assays revealed the direct binding of HOXA13 to the *ACLY* (**F**) or *IGF1R* (**G**) promoter in SW480-HOXA13 cells and the enriched binding sites of endogenous HOXA13 to the *ACLY* or *IGF1R* promoter in CRC tissues. The amounts of immunoprecipitated products were examined by real-time PCR. **H** Western blotting analysis presenting ACLY and IGF1R expression in SW480 and SW620 cells after lentivirus transfection. **I** Transwell assays showed that downregulation of ACLY and IGF1R reduced the migration and invasion abilities of SW480-HOXA13 cells, while upregulation of ACLY and IGF1R increased the migration and invasion abilities of SW620-shHOXA13 cells. **J** In vivo liver metastatic assays. Four stable cell lines were injected into the spleens of nude mice (*n* = 10 mice per group). Representative bioluminescent imaging, incidence of liver metastasis, intensity of bioluminescence signals, overall survival times, liver tissues’ HE staining in each group, and the number of metastatic liver nodules were shown. The scale bars represent 1 mm (low magnification) and 100μm (high magnification). **K** In vivo lung metastatic assays. Four stable cell lines were injected into the tail veins of nude mice (*n* = 10 mice per group). Representative bioluminescent imaging, incidence of lung metastasis, intensity of bioluminescence signals, overall survival times, lung tissues’ HE staining in each group and the number of metastatic lung nodules were shown. The scale bars represent 1 mm (low magnification) and 100μm (high magnification). *P < 0.05, **P < 0.01. Data are displayed as the mean ± s.d.

To determine the molecular mechanism underlying HOXA13-mediated CRC metastasis, human Cell Motility and Cancer PathwayFinder RT^2^ Profiler PCR Arrays were used to analyze the gene expression changes in HOXA13-overexpressing CRC cell lines (Supplementary Tables S2 and S3), since cell motility is the fundamental cellular behavior contributing to metastasis^29^. To designate differentially expressed genes in PCR arrays, we used twofold as a cut-off. Forty out of 165 genes were upregulated in SW480-HOXA13 cells than in SW480-control cells. Thirty-five out of 165 genes were elevated in DLD-1-HOXA13 cells than in DLD-1-control cells. Among the overlap of eight upregulated genes in both SW480-HOXA13 and DLD-1-HOXA13 cells (Fig. 2A), ACLY and IGF1R attracted our attention, which were strongly increased by HOXA13 overexpression. Real-time PCR and western blotting assays indicated that elevated HOXA13 markedly upregulated the expression levels of ACLY and IGF1R, whereas knockdown of HOXA13 significantly reduced ACLY and IGF1R expression levels (Fig. 2B). Luciferase reporter assays indicated that upregulation of HOXA13 elevated the *ACLY* and *IGF1R* genes’ promoter activities (Fig. 2C).

Through analyzing the sequences of *ACLY* and *IGF1R* promoters, we identified three potential HOXA13 binding sites within both *ACLY* and *IGF1R* promoters. The serial deletion assays displayed that depletion of the region of *ACLY* promoter between −1424 to −271 bp obviously abolished the HOXA13-mediated *ACLY* promoter transactivation. Accordingly, the mutation of HOXA13 binding site 2 and 1 within the *ACLY* promoter significantly inhibited the HOXA13-mediated *ACLY* promoter transactivation, whereas mutating the HOXA13 binding site 3 did not affect the *ACLY* promoter transactivation mediated by HOXA13. These studies suggested that HOXA13 binding sites 2 and 1 were essential for the *ACLY* promoter activation that was mediated by HOXA13 (Fig. 2D). Analogously, depletion of the region of *IGF1R* promoter between −1720 to −496 bp significantly abrogated HOXA13-medicated *IGF1R* promoter transactivation. Mutating HOXA13 binding site 1 within the *IGF1R* promoter dramatically inhibited the HOXA13-induced *IGF1R* promoter transactivation (Fig. 2E). Furthermore, chromatin immunoprecipitation (ChIP) assay indicated that HOXA13 bound to the promoters of both *ACLY* and *IGF1R* genes in SW480-HOXA13 cells and primary CRC tissues (Fig. 2F, G).

To further examine whether ACLY and IGF1R participated in HOXA13-induced CRC invasion and metastasis, we downregulated the expression of ACLY and IGF1R in SW480-HOXA13 cells, and upregulated the expression of these genes in SW620-shHOXA13 cells (Fig. 2H). Knocked down of ACLY and IGF1R suppressed the migratory and invasive abilities of SW480-HOXA13 cells. In contrast, ectopic overexpression of ACLY and IGF1R rescued the SW620-shHOXA13 cells’ migratory and invasive capabilities (Fig. 2I). The liver metastatic assays revealed that knocked down of ACLY and IGF1R reduced the BLI signals’ intensity, the metastatic liver nodules’ number, and the liver metastasis incidence, and extended the SW480-HOXA13 group’s overall survival times. While upregulation of ACLY and IGF1R increased the intensity of BLI signals, the metastatic liver nodules’ number, and the liver metastasis incidence, and reduced the SW620-shHOXA13 group’s overall survival times (Fig. 2J). Similar effects were identified in the lung metastatic models (Fig. 2K). Based on the above findings, ACLY and IGF1R are essential for HOXA13-induced CRC metastasis.

### IGF1-IGF1R signaling upregulates HOXA13 expression via PI3K/AKT/HIF1α pathway

**Fig. 3:**
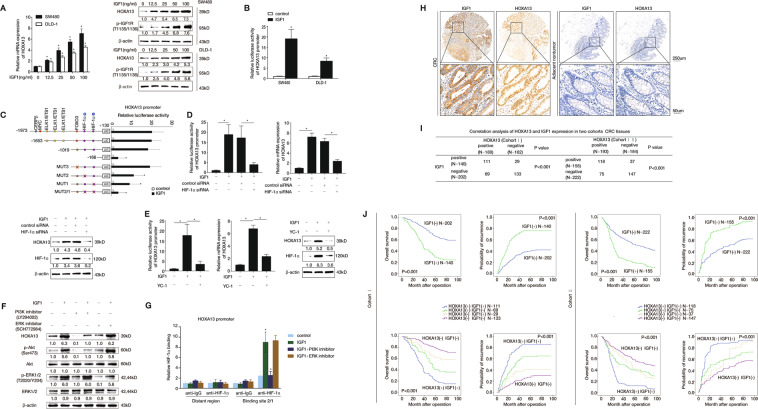
IGF1-IGF1R upregulates HOXA13 expression via PI3K/AKT/HIF1α pathway. **A** SW480 and DLD-1 cells were administrated with gradient concentrations of IGF1 for 24 h, and then the mRNA and protein levels of HOXA13 and p-IGF1R were examined by real-time PCR and western blotting. **B** Relative luciferase activities were detected in SW480 cells and DLD-1 cells, which were transfected with plasmid constructs containing *HOXA13* promoter after IGF1 treatment (50 ng/mL, 24 h). **C** Deletion and selective mutation analysis showed that two HIF1α binding regions within the *HOXA13* promoter were responsible for IGF1-induced HOXA13 promoter transactivation. **D** Knockdown of HIF1α through HIF1α siRNA reduced IGF1-mediated HOXA13 expression, which were detected by luciferase reporter assay (upper), real-time PCR (upper), and western blotting analyses (lower). **E** HIF1α inhibitor YC-1 reduced IGF1-mediated HOXA13 expression. The promoter activity (left), mRNA expression (middle), and protein expression (right) of *HOXA13* were examined when SW480 cells were treated with or without IGF1 (50 ng/mL, 24 h) in the presence of YC-1. **F** The protein expression of HOXA13, phosphorylated and total Akt and ERK1/2 were detected by western blotting when SW480 cells were pretreated with inhibitors of ERK1/2 (SCH772984) or PI3K (LY294002), and then were treated with IGF1 (50 ng/mL, 24 h) or not. **G** The ChIP assay showed the direct binding abilities of HIF1α to the *HOXA13* promoter induced by IGF1, and PI3K inhibitor reduced the binding abilities of HIF1α to the *HOXA13* promoter. **H** The expression of IGF1 and HOXA13 in CRC and adjacent nontumor tissues by IHC analysis. **I** The correlation analysis between IGF1 and HOXA13 in two independent cohorts. **J** Kaplan–Meier’s analysis showed the correlation between HOXA13/IGF1 expression and overall survival or recurrence in two independent cohorts. *P < 0.05, **P < 0.01. Data are displayed as the mean ± s.d.

Since elevated expression of HOXA13 promoted CRC metastasis, the concrete regulatory mechanism of HOXA13 overexpression in CRC needs to be elucidated. IGF1R participated in HOXA13-mediated CRC metastasis. Thus, we focused on its ligand IGF1. IGF1, as a major mediator of the effects of the growth hormone, is beneficial to cell proliferation and suppressing apoptosis^30,31^. Overexpression of IGF1 promotes CRC initiation and progression, while inhibiting IGF1 can impede CRC progression^28^. In view of the significant functional roles of IGF1-IGF1R signaling and HOXA13 in CRC metastasis, the hypothesis of whether IGF1-IGF1R signaling regulated HOXA13 expression was proposed. SW480 and DLD-1 cells that have a low endogenous expression of HOXA13 were administrated with recombinant human IGF1. Treatment of IGF1 elevated the mRNA and protein expression of HOXA13, and the protein expression of p-IGF1R in a dose-dependent manner (Fig. 3A). To further explore whether IGF1-mediated HOXA13 overexpression was induced through the transactivation of its promoter, we administrated SW480 and DLD-1 cells with IGF1 after transfection with *HOXA13* promoter construct. The luciferase activity of *HOXA13* promoter was distinctly elevated after IGF1 treatment (Fig. 3B).

To explore the function of cis-regulatory elements of *HOXA13* promoter involved in IGF1 stimulation, the region of −1999 to +130 bp, and a sequence of truncated mutants of *HOXA13* promoter was developed. A marked decline in *HOXA13* promoter activity was detected after SW480 cells were transfected with the truncated (−1019 to −166) HOXA13 promoter, demonstrating that this sequence, in which two HIF1α potential binding sites and one FOXO3 potential binding site were located, was essential for the activation of *HOXA13* promoter induced by IGF1. Subsequently, site-directed mutagenesis discovered that mutation of HIF1α binding sites obviously reduced *HOXA13* promoter activity, although mutation of the FOXO3 binding site had no obvious effect on *HOXA13* promoter activity (Fig. 3C). Knockdown of HIF1α obviously suppressed the enhanced *HOXA13* promoter activity, and reduced the mRNA and protein expression of IGF1-induced HOXA13 overexpression (Fig. 3D). Furthermore, YC-1, HIF1α inhibitor, also significantly reduced IGF1-mediated *HOXA13* promoter transactivation and HOXA13 overexpression (Fig. 3E). Besides, the function of HIF1α in HOXA13-induced CRC metastasis was detected. SW480 cells were transfected with lentiviral vectors to construct HIF1α-overexpressing SW480 cells (SW480-HIF1α), and HOXA13 expression was further knockdown via lentiviral transfection in SW480-HIF1α cells. Transwell assays revealed that overexpression of HIF1α enhanced the migratory and invasive capabilities of SW480 cells, while HOXA13 knockdown impaired the enhanced migratory and invasive abilities of SW480-HIF1α cells (Supplementary Fig. S1).

Since IGF1-IGF1R signaling activates PI3K/AKT and MEK/ERK pathways^21^, we determined which pathway regulates IGF1-induced HOXA13 upregulation. CRC cells were administrated with PI3K inhibitor (LY294002) and ERK inhibitor (SCH772984). Treating with PI3K inhibitor significantly decreased IGF1-induced HOXA13 expression, however, administrating with ERK inhibitor had no obvious effect (Fig. 3F). Moreover, ChIP analysis found that treatment with PI3K inhibitor markedly suppressed the binding of HIF1α to the *HOXA13* promoter that was induced by IGF1, whereas treatment with ERK inhibitor had no significant effect (Fig. 3G).

In addition, to explore the clinical relationship between IGF1 and HOXA13, IHC staining was then used in two CRC cohorts. The IGF1 expression level was markedly elevated in primary CRC than adjacent nontumor specimens, and showed a positive correlation with HOXA13 expression (Fig. 3H, I). Moreover, IGF1 expression had positive effect on lymph node metastasis, distant metastasis, worse tumor differentiation, and higher AJCC stage (Supplementary Table S4). CRC patients who had positive IGF1 expression showed lower overall survival times and higher recurrence rate than those who had negative IGF1 expression in both cohorts (Fig. 3J, upper panel). Further, CRC patients who had positive co-expression of IGF1 and HOXA13 exhibited the lowest overall survival and highest recurrence rates in both CRC cohorts (Fig. 3J, lower panel).

### HOXA13 is critical for IGF1-induced CRC metastasis

**Fig. 4:**
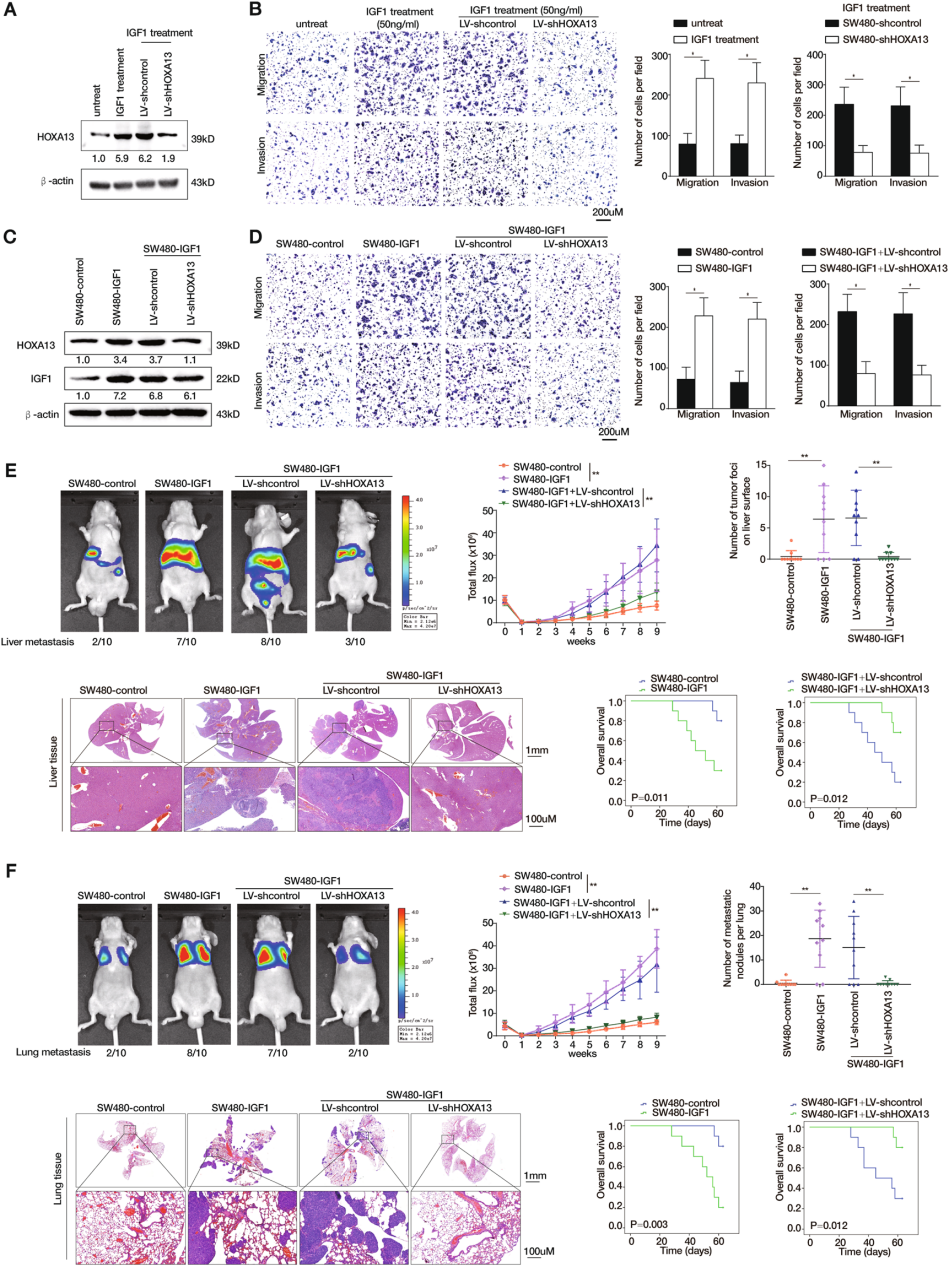
HOXA13 is critical for IGF1-induced CRC metastasis. **A** SW480 cells were transfected with LV-shcontrol or LV-shHOXA13 lentiviral vectors and then treated with or without IGF1 (50 ng/mL, 24 h). Next, the HOXA13 protein expression was analyzed by western blotting. **B** Transwell assays demonstrated that IGF1 treatment increased the migratory and invasive abilities of SW480 cells, while HOXA13 knockdown reduced these capabilities of SW480 cells. **C** SW480 cells were transfected with lentiviral vectors to construct IGF1-overexpressing SW480 cells (SW480-IGF1), and HOXA13 expression was further knockdown via lentiviral transfection in SW480-IGF1 cells. IGF1 and HOXA13 expression in the indicated cell lines was examined by western blotting. **D** Transwell assays revealed that overexpression of IGF1 enhanced the migratory and invasive capabilities of SW480 cells, while HOXA13 knockdown impaired the enhanced migratory and invasive abilities of SW480-IGF1 cells. **E** In vivo liver metastatic assays. Four stable cell lines were injected into the spleens of nude mice (*n* = 10 mice per group). Representative bioluminescent imaging, intensity of bioluminescence signals, incidence of liver metastasis, liver tissues’ HE staining, number of metastatic liver nodules, and overall survival times in each group were shown. The scale bars represent 1 mm (low magnification) and 100 μm (high magnification). **F** In vivo lung metastatic assays. Four stable cell lines were injected into the tail veins of nude mice (*n* = 10 mice per group). Representative bioluminescent imaging, intensity of bioluminescence signals, incidence of lung metastasis, lung tissues’ HE staining, number of metastatic lung nodules, and overall survival times in each group (*n* = 10) were shown. The scale bars represent 1 mm (low magnification) and 100 μm (high magnification). *P < 0.05, **P < 0.01. Data are displayed as the mean ± s.d.

In view of the upregulated expression of HOXA13 treated by IGF1 and the pivotal role of HOXA13 in promoting CRC metastasis, we subsequently explored whether HOXA13 was involved in IGF1-mediated CRC metastasis. SW480 cells were transducted with LV-shHOXA13 lentivirus to knockdown HOXA13, and then were administrated with 50 ng/mL IGF1 for 24 h. Next, the HOXA13 protein expression in SW480 and SW480-shHOXA13 cells treated with or without IGF1 was examined by western blotting (Fig. 4A). Transwell assays demonstrated that IGF1 treatment promoted the migratory and invasive abilities of SW480 cells, while downregulation of HOXA13 reduced the enhanced migratory and invasive capabilities of SW480 cells induced by IGF1 treatment (Fig. 4B). Subsequently, a SW480-IGF1 stable cell line was constructed, and SW480-IGF1 cells were infected with LV-shHOXA13 to downregulate HOXA13 expression. Western blotting assays were conducted to evaluate the protein levels of HOXA13 and IGF1 in the indicated cell lines (Fig. 4C). Elevated expression of IGF1 upregulated the migratory and invasive capabilities of SW480 cells, whereas HOXA13 knockdown decreased the above-enhanced capabilities of SW480-IGF1 cells (Fig. 4D).

Further, the liver metastatic assays revealed that increased IGF1 expression upregulated the BLI intensity and the liver metastasis incidence with its metastatic nodules’ number, and reduced the SW480-control xenograft group’s overall survival. Nevertheless, HOXA13 knockdown reduced the BLI intensity and the liver metastasis incidence with its metastatic liver nodules’ number, and increased the SW480-IGF1 xenograft group’s overall survival times (Fig. 4E). Similar effects were identified in the lung metastatic models (Fig. 4F). Thus, HOXA13 plays a critical role in IGF1-mediated CRC metastasis.

### HOXA13 expression had a positive correlation with ACLY and IGF1R expression in CRC tissues

**Fig. 5:**
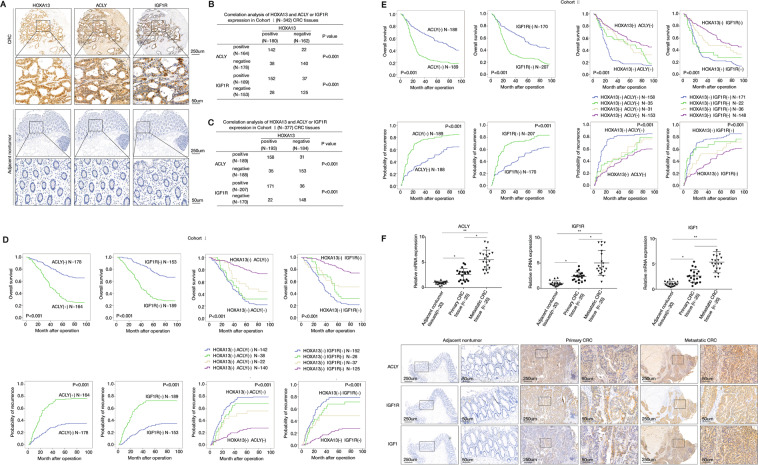
HOXA13 expression has a positive correlation with ACLY and IGF1R expression in CRC tissues. **A** Representative images of IHC staining of HOXA13, ACLY, and IGF1R expression in human CRC and adjacent nontumor samples were shown. The scale bars represent 250 μm (low magnification) and 50 μm (high magnification). **B**, **C** Correlation analysis of HOXA13 and ACLY or IGF1R expression in the CRC tissues in cohort I (**B**) and cohort II (**C**). **D**, **E** Kaplan–Meier’s curves generated with the data from the CRC patients with negative versus positive ACLY or IGF1R expression. The correlation between HOXA13 and ACLY (left) or IGF1R (right) expression and overall survival or recurrence in patients with CRC in cohort I (**D**) and cohort II (**E**). **F** Real-time PCR (upper) and IHC analyses (lower) of ACLY, IGF1R, and IGF1 expression in adjacent nontumor samples, primary CRC, and paired metastasis CRC samples. The scale bars represent 250 μm (low magnification) and 50 μm (high magnification). *P < 0.05, **P < 0.01. Data are displayed as the mean ± s.d.

The expression of ACLY and IGF1R were analyzed in two independent CRC cohorts. IHC staining showed that both ACLY and IGF1R expression were significantly elevated in primary CRC than adjacent nontumor specimens. The expression of ACLY and IGF1R showed a positive correlation with HOXA13 expression in two CRC cohorts (Fig. 5A–C). The overexpression of ACLY and IGF1R was correlated with a higher risk of lymph node and distant metastasis, worse tumor differentiation, and higher AJCC stage (Supplementary Tables S5 and S6). In addition, Kaplan–Meier analysis exhibited that CRC patients who had a positive expression of ACLY or IGF1R exhibited higher recurrence rate and lower overall survival times than those who had a negative expression of ACLY or IGF1R in both cohorts (Fig. 5D, E). Moreover, CRC patients who had positive co-expression of either HOXA13/ACLY or HOXA13/IGF1R showed the lowest survival times and highest recurrence rates in both CRC cohorts (Fig. 5D, E). To further investigate the roles of ACLY, IGF1R, and IGF1 in CRC metastasis, the expression of ACLY, IGF1R, and IGF1 were examined in 20 pairs of primary CRC, metastatic CRC, and adjacent noncancerous tissues. Real-time PCR and IHC staining indicated that the expression of ACLY, IGF1R, and IGF1 were evidently higher in metastatic CRC than in primary CRC and adjacent noncancerous samples (Fig. 5F).

### Combination of ACLY inhibitor and IGF1R inhibitor dramatically decreases HOXA13-mediated CRC metastasis

**Fig. 6:**
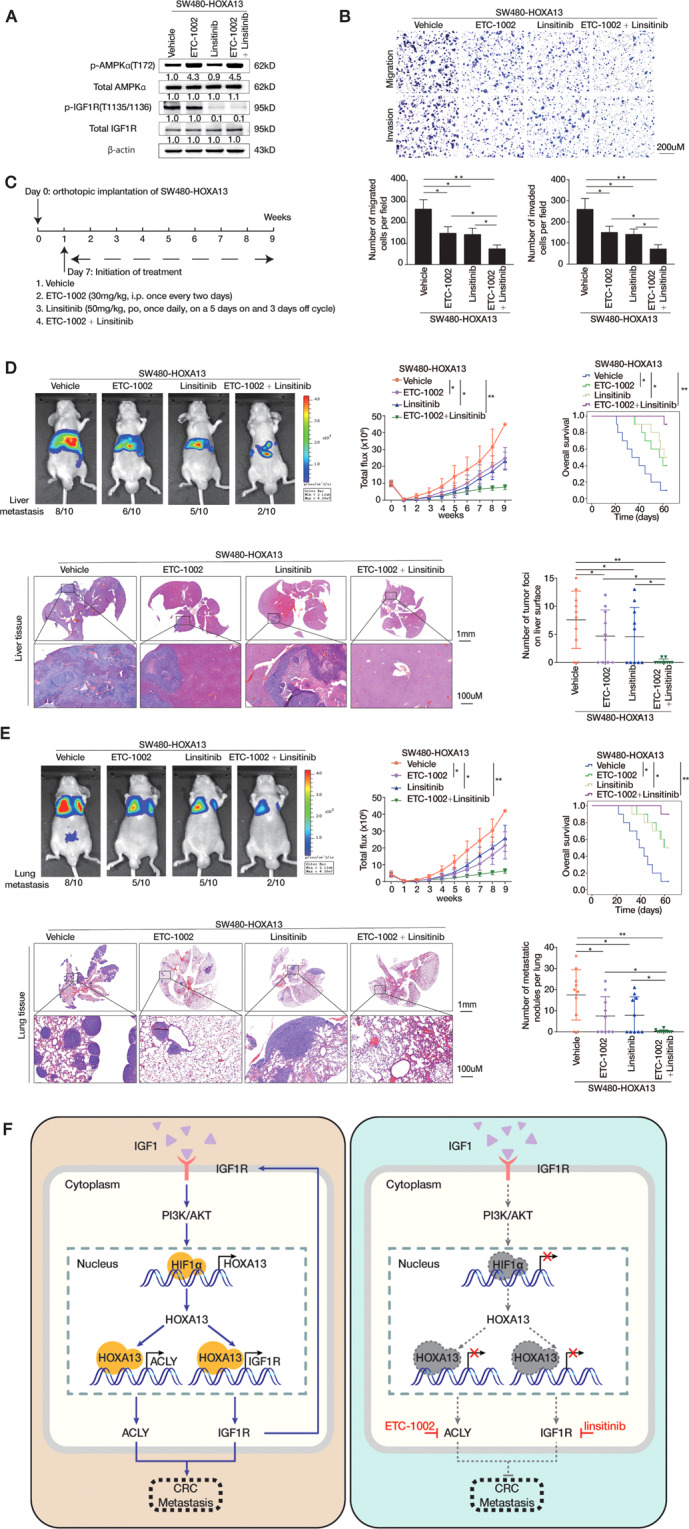
Combination of ACLY inhibitor and IGF1R inhibitor dramatically decreases HOXA13-mediated CRC metastasis. **A** The SW480-HOXA13 cells were respectively treated with ACLY inhibitor ETC-1002 (100 μM, 12 h), IGF1R inhibitor Linsitinib (10 μM, 6 h) or both two agents. The protein expression levels of p-AMPKα, total AMPKα, p-IGF1R, and total IGF1R were detected by western blotting. **B** Transwell assays showed the migratory and invasive capabilities of SW480-HOXA13 cells after treatment with ACLY inhibitor ETC-1002 (100 μM, 12 h), IGF1R inhibitor Linsitinib (10 μM, 6 h) or the combination of both two agents. **C** The diagram of agent treatment in four randomly assigned groups of nude mice. These mice were treated with vehicle, ETC-1002 (30 mg/kg), Linsitinib (50 mg/kg) or combined treatment 1 week after injection of SW480-HOXA13 cells. **D** In vivo liver metastatic assays revealed that combined administration of ETC-1002 and Linsitinib significantly suppress HOXA13-mediated CRC metastasis. Four stable cell lines were injected into the spleens of nude mice (*n* = 10 mice per group). Representative bioluminescent imaging, intensity of bioluminescence signals, incidence of liver metastasis, liver tissues’ HE staining, number of metastatic liver nodules, and overall survival times in each group were shown. The scale bars represent 1 mm (low magnification) and 100 μm (high magnification). **E** In vivo lung metastatic assays revealed that combined administration of ETC-1002 and Linsitinib significantly suppress HOXA13-mediated CRC metastasis. Four stable cell lines were injected into the tail veins of nude mice (*n* = 10 mice per group). Representative bioluminescent imaging, intensity of bioluminescence signals, number of metastatic lung nodules, overall survival times, lung tissues’ HE staining in each group, and the incidence of lung metastasis were shown. The scale bars represent 1 mm (low magnification) and 100 μm (high magnification). **F** A schematic diagram for the role of IGF1-HOXA13-IGF1R positive feedback loop in CRC metastasis. IGF1-IGF1R pathway upregulates HOXA13 via PI3K/AKT/HIF1α pathway. HOXA13 promotes CRC metastasis through upregulating ACLY and IGF1R expression. Combined administration of ACLY inhibitor (ETC-1002) and IGF1R inhibitor (Linsitinib) disrupts the IGF1-HOXA13-IGF1R loop and inhibits CRC metastasis. *P < 0.05, **P < 0.01. Data are displayed as the mean ± s.d.

Bempedoic Acid (ETC-1002) as an ACLY inhibitor has been approved by the Food and Drug Administration (FDA) for decreasing LDL-C levels in atherosclerotic cardiovascular disease, and has been applied to cancer therapeutic therapy^32^. Linsitinib (OSI-906) as IGF1R inhibitor suppresses tumor growth and invasion by decreasing cell viability and inducing apoptosis^23^. Based on our above finding that IGF1/IGF1R axis facilitated HOXA13 overexpression and CRC metastasis through transactivating ACLY and IGF1R, we hypothesized that whether the combination of ACLY and IGF1R inhibitors had a synergistic effect on CRC treatment. Firstly, SW480-HOXA13 cells were treated with ETC-1002 or Linsitinib, or both agents. The protein expression levels of p-AMPKα, total AMPKα, p-IGF1R, and total IGF1R were detected by western blotting (Fig. 6A). Either ETC-1002 or Linsitinib treatment moderately decreased the migratory and invasive capabilities of SW480-HOXA13 cells. Combined treatment of two agents distinctly reduced the migratory and invasive abilities of SW480-HOXA13 cells (Fig. 6B). Further, the endogenous expression of HOXA13 in SW620 cells treated with Linsitinib was detected by western blotting. We found that treatment of IGF1R inhibitor Linsitinib reduced the protein expression of HOXA13 (Supplementary Fig. S2).

Then, in vivo metastatic experiments were performed (Fig. 6C). The liver and lung metastatic assays showed that either ETC-1002 or Linsitinib administration partially reduced the intensity of BLI signals, the metastatic nodules’ number, the incidence of metastasis, and extended SW480-HOXA13 group’s overall survival times. However, when compared with the control xenograft group, administration of ETC-1002 and Linsitinib markedly reduced the intensity of BLI signals, the metastatic nodules’ number, the incidence of metastasis, and largely prolonged overall survival times (Fig. 6D, E). These findings demonstrated that combined administration of ACLY inhibitor ETC-1002 and IGF1R inhibitor Linsitinib significantly inhibited HOXA13-mediated CRC metastasis (Fig. 6F).

## Discussion

Metastasis is still a principal element of poor clinical outcomes among CRC patients^2^. Thus, exploring the complicated metastatic mechanisms would be of great significance for targeted therapy. ATP-citrate lyase (ACLY), which principally located in cytosol and nucleus, is a rate-controlling enzyme catalyzing the conversion of citrate and coenzyme A (CoA) to oxaloacetate and acetyl-CoA, the latter one functioning as a substrate for fatty acid and cholesterol synthesis^33^. ACLY, thus, occupies a privileged position linking lipogenesis and glucose catabolism^34^. Lipogenesis and glycolysis are indispensable metabolic processes for tumor progression and metastasis^35^. Several studies reported that elevated ACLY expression is involved in the initiation, progression, and metastasis in many aggressive cancers by accelerating lipid synthesis and tumor progression, including CRC^34,36^. Increased expression of ACLY is responsible for the superior ability of metastasizing to liver and lung, and inhibition of ACLY reduces the abilities of EMT and metastasis in colon cancer^36^. In addition, upregulated ACLY has been reported in chemo-resistant CRC cells, and inhibition of ACLY re-sensitized the cells to SN38^37^. Thus, ACLY is a promising target in CRC. As mentioned earlier, IGF1R functions as an oncogene and is regarded as a key determinant of CRC development and metastasis^24^. Therefore, IGF1R may be developed as a potential target for CRC therapy. Taken together, ACLY and IGF1R are key factors in CRC progression and metastasis. In the current study, we discovered that HOXA13 transactivated the expression of ACLY and IGF1R. Knockdown of ACLY or IGF1R abrogated HOXA13-enhanced CRC metastasis, while upregulated expression of ACLY or IGF1R rescued the decreased CRC metastatic ability induced by knockdown of HOXA13. Furthermore, in human CRC samples, HOXA13 presented a positive correlation with the expression of ACLY and IGF1R. Patients with positive co-expression of HOXA13/ACLY or HOXA13/IGF1R displayed the worst prognosis. Therefore, HOXA13 facilitated CRC progression and metastasis through upregulating ACLY and IGF1R expression.

We further explored the regulatory mechanism of elevated HOXA13 expression in CRC. IGF1R is a well-established therapeutic target in CRC, and it has high affinity to IGF1 ligand. IGF1, as a major mediator of the effects of the growth hormone, is beneficial to cell proliferation and suppressing apoptosis^30,31^. Importantly, several studies have reported that IGF1-IGF1R signaling contributes to CRC cell survival, proliferation, invasion, metastasis, and chemotherapeutic resistance^24,25^. In the present study, we found that IGF1-IGF1R signaling upregulated the expression level of HOXA13 via PI3K/AKT/HIF1α pathway. Moreover, IGF1-induced HOXA13 overexpression upregulated IGF1R expression, which facilitated the CRC sensitivity to IGF1 stimulation and formed an IGF1-HOXA13-IGF1R positive feedback loop. Interestingly, knockdown of HOXA13 reduced IGF1-mediated CRC metastatic ability. Therefore, targeting this positive feedback loop may provide a potential treatment strategy to inhibit CRC metastasis.

In consideration of ACLY and IGF1R as promising therapeutic targets, we further designed a pharmacological strategy to verify their crucial anti-cancer roles. ACLY inhibitor ETC-1002 has been approved by the FDA for reducing cholesterol synthesis and LDL-C level, and has attracted interests as a potential anti-cancer agent^32^. Previous studies reported that combined treatment of ETC-1002 and anti-PD-L1 markedly reduced liver tumorigenesis^38^. However, few studies report the effect of ETC-1002 in CRC treatment. Moreover, there are three main strategies targeting IGF1R: tyrosine kinase inhibitors, IGF1R antibodies that inducing its internalization and degradation, and neutralizing antibodies that targeting its ligand^39,40^. Among the above strategies, targeting IGF1R with antibodies has been widely used in clinical trials, however, the responses were generally low^41^. Thus, tyrosine kinase inhibitors stand out. Linsitinib (OSI-906), a selective IGF1R tyrosine kinase inhibitor, can suppress tumor growth and invasion^23^. Studies showed that IGF1R inhibitor Linsitinib could suppress tumor progression in many cancer types^42,43^, including CRC^44^. Based on the above understanding, we hypothesized that whether the combined application of ACLY inhibitor ETC-1002 and IGF1R inhibitor Linsitinib had any effect on CRC metastasis. In this study, we found that there was only a modest effect when using ETC-1002 or Linsitinib alone, while the combination of ETC-1002 and Linsitinib attained dramatic synergy in the suppression of HOXA13-induced CRC metastasis. Therefore, combination therapy of ACLY inhibitor and IGF1R inhibitor might emerge as a promising therapeutic strategy in HOXA13-induced CRC metastasis.

In summary, we indicated that IGF1 upregulated HOXA13 expression, and HOXA13 upregulated ACLY and IGF1R expression and facilitated CRC metastasis. The combined administration of ACLY inhibitor and IGF1R inhibitor significantly disrupted the IGF1-HOXA13-IGF1R feedback loop and suppressed HOXA13-induced CRC metastasis. Therefore, HOXA13 is a potential prognostic biomarker, and targeting the IGF1-HOXA13-IGF1R oncogenic loop may provide a potential therapeutic strategy to inhibit HOXA13-driven CRC metastasis.

## Materials and methods

### Cell culture

All CRC cell lines used in this study were obtained from the American Tissue Type Culture Collection (Rockville, MD). Each cell line was tested and authenticated by their manufacturers. The cells were cultured in Dulbecco’s modified Eagle’s medium (Gibco, ThermoFisher Scientific, Cambridge, MA, USA) supplemented with 10% fetal bovine serum (FBS, Gibco), 100 μg/mL penicillin, and 100 μg/mL streptomycin (Gibco) in a 5% CO_2_ atmosphere at 37 °C. These above cell lines were authenticated by short tandem repeats (STRs) DNA profiling. All cells were tested for mycoplasma contamination before use with the Universal Mycoplasma Detection Kit (ATCC 30–1012K) and were not contaminated by mycoplasma.

### Patients and follow-up

Written informed consent was obtained from each patient, and ethical approval was obtained from the Ethics Committee of the Fourth Military Medical University and Tongji Medical College. Cohort I included freshly sampled CRC tissues with healthy adjacent tissues collected between January 2005 and December 2007 from 342 adult patients who underwent surgery at Xijing Hospital of the Fourth Military Medical University (Xi’an, China). Cohort II included CRC tissue samples that were surgically resected from 377 adult CRC patients between January 2005 and December 2007 at the Tongji Hospital of Tongji Medical College (Wuhan, China). All patients were staged pathologically based on the American Joint Committee on Cancer (AJCC)/International Union against Cancer criteria. All patients were preoperative radiotherapy- and chemotherapy-naive; however, those with stage II–IV disease received postoperative adjuvant chemotherapy. No patients were treated with postoperative radiotherapy. Primary tumor samples along with dissected regional lymph nodes were subjected to histomorphological analysis via hematoxylin–eosin (H&E) staining performed by the Department of Pathology of Xijing and Tongji Hospital. HOXA13 mRNA expression was assessed in 120 pairs of frozen fresh CRC tissues and adjacent nontumor tissues that were collected during surgical resection and frozen in liquid nitrogen.

The information collected during the follow-up period included the incidence of disease recurrence and the presence of distant metastasis as confirmed by imaging and procedural data (positron emission tomography, ultrasonography, magnetic resonance imaging, computed tomography, and endoscopy) or pathological data (biopsies and cytologic analysis). Overall survival time was defined as the period between surgical resection and death. The duration of disease-free survival was defined as the period between surgical resection and the emergence of either distant CRC metastasis or CRC recurrence, the occurrence of another noncolorectal cancer (with the exception of carcinoma in situ of the cervix and skin basal cell carcinoma) or death from any cause without documentation of a cancer-related event. Patients were followed up for a minimum of 8 years, with follow-up data collected via questionnaire letters and telephone inquiry; patient databases were updated every 3 months. Patient deaths were determined by a corroborative history from the family and verified by reviewing public records.

### Plasmid construction

The HOXA13 gene complete CDS construct, pCMV-HOXA13, was generated by using cDNA from human PBMCs. It was generated with forward and reverse primers incorporating EcoRI and XhoI sites at the 5′ and 3′-ends, respectively. The polymerase chain reaction (PCR) product was cloned into the EcoRI and XhoI sites of the pCMV-Tag2B vector. Human genomic DNA was applied to the ACLY promoter construct (−1910/+109) ACLY, which corresponds to the −1910 to +109 sequence (relative to the transcriptional start site) of the 5′-flanking region of ACLY gene. Forward and reverse primers containing the *Nhe*l and *Xho*I sites at the 5′ and 3′-ends, respectively, were performed to build the construct. The final PCR product was cloned into the *Nhe*l and *Xho*I sites of the pGL3-Basic vector (Promega). Analogously, constructs containing a deletion of the 5′-flanking region of the ACLY promoter, [(−1424/+109) ACLY, (−559/+109) ACLY, and (−271/+109) ACLY], were built and the (−1910/+109) ACLY construct served as the template. The HOXA13 binding sites of the ACLY promoter were mutated by the QuikChange II Site-Directed Mutagenesis Kit (Stratagene, CA, USA). Gene sequencing was used to confirm the sequence integrity of all constructs. All promoter constructs used in the experiment were generated similarly. All primers are listed in Supplementary Table S1.

### Transient transfection

A total of 1 × 10^5^ serum-starved cells were plated in each well of a 24-well plate and allowed to attach for 12–24 h. Then, a mixture of Lipofectamine 2000 (Invitrogen, USA) containing 0.02 μg of the pRL-TK plasmids, 0.18 μg of the promoter reporter plasmids, and 0.6 μg of the expression vector plasmids was used to co-transfect cells for 5 h based on the manufacturer’s instructions. After 5 h of transfection, the cells were then washed and incubated with 1% FBS-supplemented fresh medium for 48 h.

### Luciferase reporter assay

The Dual Luciferase Assay (Promega, USA) was used to quantify luciferase activity according to the manufacturer’s instructions. Transfected cells were lysed in a culture dish with lysis buffer, and the resulting lysates were centrifuged at maximum speed for 1 min in an Eppendorf microcentrifuge. The efficiency of transfection was normalized to Renilla activity, and relative luciferase activity was quantified with a Modulus^TM^ TD20/20 Luminometer (Turner Biosystems, USA).

### Western blotting analyses

Proteins from lysed cells were fractionated by SDS-PAGE and transferred to nitrocellulose membranes. Nonspecific binding sites were blocked with 5% milk in TBST (120 mM Tris–HCl (pH 7.4), 150 mM NaCl, and 0.05% Tween 20) for 1 h at room temperature. Blots were incubated with a specific antibody overnight at 4 °C. Western blotting of β-actin on the same membrane was used as a loading control. The membranes were incubated with primary antibodies overnight at 4 °C. The membranes were then washed with PBS three times and incubated with an HRP-conjugated secondary antibody. Proteins were visualized using a Immobilon^TM^ Western Chemiluminescent HRP substrate (Millipore, USA). The primary antibodies used are listed below: anti-HOXA13 (ab106503) and anti-ACLY (ab40793) were purchased from Abcam. Anti-IGF1R (3027), anti-p-IGF1R (3024), anti-HIF1α (36169), anti-AMPKα (5831), anti-p-AMPKα (2535), anti-p-ERK1/2(T202/Y204) (4370), anti-ERK1/2 (9102), anti-p-Akt (Ser-473) (4060), and anti-Akt (4685) were purchased from Cell Signaling Technology. Anti-β-actin (A1978) and anti-IGF1 (DF6090) were purchased from Sigma and Affinity, respectively.

### Real-time PCR

The RNeasy Plus Mini Kit (50) kit (Qiagen, Hilden, Germany) was used to extract total RNA, which was then reverse transcribed with the Advantage RT-for-PCR Kit (Qiagen) in accordance with the manufacturer’s protocols. The target sequence was amplified with real-time PCR with the SYBR Green PCR Kit (Qiagen). The cycling parameters used were 95 °C for 15 s, 55–60 °C for 15 s, and 72 °C for 15 s for 45 cycles. Melting curve analyses were performed, and Ct values were determined during the exponential amplification phase of real-time PCR. SDS 1.9.1 software (Applied Biosystems, Massachusetts, USA) was used to evaluate amplification plots. The 2^–ΔΔCt^ method was used to determine relative fold changes in target gene expression in cell lines, which was normalized to expression levels in corresponding control cells (defined as 1.0). The equation used was 2^–ΔΔCt^ (ΔCt = ΔCt^target^ – ΔCt^GAPDH^; ΔΔCt = ΔCt^expressing vector^ – ΔCt^control vector^). When calculating relative expression levels in surgically extracted CRC samples, relative fold changes in target gene expression were normalized to expression values in normal colon epithelial tissues (defined as 1.0) using the following equation: 2 ^–ΔΔCt^ (ΔΔCt = ΔCt^tumor^ – ΔCt^nontumor^). All experiments were performed in duplicate. Supplementary Table S1 lists all sequences of all primers used.

### Chromatin immunoprecipitation assay (ChIP)

Cells were immersed in 1% formaldehyde for 10 min at 37 °C to stimulate cross-linking. Then, glycine was used to quench the formaldehyde after cross-linking to stop formaldehyde fixation. After washing with PBS, the cells were resuspended in lysis buffer (1 mM PMSF, 1% SDS, 10 mM EDTA, and 50 mM Tris (pH 8.1) – total volume 300 μl). Sonication was then performed to produce fragmented DNA. A slurry of protein G-Sepharose and herring sperm DNA (Sigma-Aldrich) was used to clear the supernatant. The recovered supernatant was then subjected to a 2-h incubation period with specific antibodies or an isotype control IgG in the presence of protein G-Sepharose beads and herring sperm DNA, followed by antibody denaturation with 1% SDS in lysis buffer. Precipitated DNA was extracted from the beads by immersing them in a 1.1 M NaHCO_3_ solution and 1% SDS solution at 65 °C for 6 h. Immunoprecipitated DNA was retrieved from the beads by immersion in 1% SDS and a 1.1 M NaHCO_3_ solution at 65 °C for 6 h. The DNA was then purified using a PCR Purification Kit (QIAGEN, USA). The primers are shown in Supplementary Table S1.

For ChIP assays of tissues, cells were first separated from six pairs of fresh frozen CRC tissues and normal colon epithelial tissues collected after surgical resection. In detail, surgically extracted tumor tissues were first washed by 1× cold, PBS, 5 min, for three times and added to medium supplemented with antibiotic and antifungal agents. Use a clean razor blade to cut a pie of tissue (around 5 mm^3^) into a small piece (typical 1 mm^3^ or smaller). Then, digestion the tissues with DNase I (20 mg/mL; Sigma-Aldrich) and collagenase (1.5 mg/mL; Sigma-Aldrich) and placed on a table concentrator, 37 °C, for 1 h. At the end of the hour, we filtered the dissociated cells through 100-μm-pore filters rinsed with fresh media. The 1× red cell lysis was added to the tissues and incubated for 5 min to lysis the red blood cell, followed by another rinse. The dissociated cells were cross-linked using 1% formaldehyde for 10 min at 37 °C. After cell lysis, the DNA was fragmented by sonication. ChIP grade antibody or IgG (negative control) was used to immunoprecipitated the fragment DNA. Then, RT-PCR was used to amplify the corresponding binding site on the promoters.

### Construction of tissue microarrays and immunohistochemistry

A tissue microarray was constructed with the sampled human CRC tissues and their respective adjacent healthy tissues (Shanghai Biochip Co., Ltd. Shanghai, China). The tissue microarray was stained for HOXA13 (Abcam, ab106503), ACLY (Abcam, ab40793), IGF1R (Cell signaling technology, 3027), and IGF1 (Affinity, DF6096) expression. The array was scored independently by two pathologists for both the staining intensity and the extent of the protein expression across the section.

Immunohistochemistry was performed on 4-μm-thick, routinely processed paraffin-embedded sections. Briefly, after baking on a panel at 60 °C for an hour, the tissue sections were deparaffinized with xylene and rehydrated through gradient ethanol immersion. Endogenous peroxidase activity was quenched by 3% (vol/vol) hydrogen peroxide in methanol for 12 min, followed by three 3-min washes with phosphate-buffered saline (PBS). Then the slides were immersed in 0.01 mol/L citrate buffer solution (pH 6.0) and placed in a microwave oven for 30 min. After washing in PBS (pH 7.4, 0.01 mol/L), the sections were incubated in a moist chamber at 4 °C overnight with the primary antibody diluted in PBS containing 1% (wt/vol) bovine serum albumin. Negative controls were performed by replacing the primary antibody with preimmune mouse serum. After three 5 min washes with PBS, the sections were treated with a peroxidase-conjugated second antibody (Santa Cruz) for 30 min at room temperature, followed by additional three 5 min washes with PBS. The reaction product was visualized with diaminobenzidine for 2 min. Images were obtained under a light microscope (Olympus, Japan) equipped with a DP70 digital camera.

Analyses were performed by two independent observers who were blinded to the clinical outcome. The immunostaining intensity was scored on a scale of 0–3: 0 (negative), 1 (weak), 2 (medium), or 3 (strong). The percentage of positive cells was evaluated on a scale of 0–4: 0 (negative), 1 (1–25%), 2 (26–50%), 3 (51–75%), or 4 (76–100%). The final immuno-activity scores were calculated by multiplying the above two scores, resulting an overall score that ranges from 0 to 12. Each case was ultimately considered “negative” if the final score ranges from 0 to 3, and “positive” if the final score ranges from 4 to 12.

### In vitro migration and invasion assays

A 24-well transwell plate (8-µm pore size, Corning, USA) was used to measure the migratory and invasive ability of each tested cell line. For transwell migration assays, 5 × 10^4^ cells were plated in the top chamber lined with a non-coated membrane. For invasion assays, chamber inserts were coated with 200 mg/mL of Matrigel and dried overnight under sterile conditions. Then, 1 × 10^5^ cells were plated in the top chamber. The mean of triplicate assays for each experimental condition was used. The average number of cells in five fields per membrane was counted in triplicate inserts. The relative invasion/migration was expressed as the number of treated cells to control cells.

### The Cell Motility and Cancer PathwayFinder RT^2^ profiler PCR array

SW480 cells were divided into two groups, namely SW480-HOXA13 and SW480-control cells. Similarly, DLD-1 cells were divided into DLD-1-HOXA13 and DLD-1-control cells. RNA extraction, DNase treatment, and RNA cleanup were conducted according to the manufacturer’s protocol (Qiagen). The cDNA of each group was synthesized using the RT^2^ First Strand Kit (Qiagen). Gene expression profiling of SW480-HOXA13 and SW480-control cells, or DLD-1-HOXA13 and DLD-1-control cells were conducted using the Human Cell Motility and Cancer PathwayFinder RT^2^ Profiler PCR Array, which represents 84 genes known to be involved in cell motility and cancer pathway, respectively. The cDNA synthesis reaction was mixed with 2× RT^2^ PCR SYBR Green Mastermix and ddH_2_O, and then dispensed to the PCR array 96-well plate (25 μL/well). A 2-step cycling program was performed using the Bio-Rad CFX96. Data normalization was done by correcting all Ct values based on the average Ct values of several housekeeping genes present on the array. Each experiment was performed in triplicate.

### In vivo metastatic model and bioluminescence imaging

Six-week-old BALB/C nude mice were cared for and maintained based on our institution’s protocols for ethical animal care. The Committee on the Use of Live Animals in Teaching and Research (CULATR) of the Tongji Medical College and Fourth Military Medical University approved all animal experiments. Mice were randomly assigned into experimental or control groups, blinding was not possible. In the tail vein injection-based in vivo metastasis assays, 10 mice in each group received tail vein injections of 1 × 10^6^ cells in 100 μL of phosphate-buffered saline (PBS). In the intrasplenic injection-based in vivo metastasis assays, the mice were first anesthetized by intraperitoneal injection (0.01 mL/mg) of a mixture of Zoletil (30 mg/kg) and Rompun (10 mg/kg). Spleens were exteriorized via a small left abdominal flank incision. A single intrasplenic injection of 2×10^6^ luciferase-labeled cells in 100 μL of Hank’s balanced salt solution (HBSS) (Gibco) was administered with a 30-gauge needle. Gentle pressure was applied to the injection site with a cotton swab for 1 min to staunch bleeding and to prevent leakage of tumor cells. Spleens were carefully reinserted into the abdominal cavity, and the wound was sutured using 4–0 black silk (10 mice per group). Every week, the mice received intraperitoneal injections of 150 mg/kg of d-luciferin, and images were acquired 10 min after injection with an IVIS 100 Imaging System (Xenogen, Hopkinton, MA, USA). Each image was acquired within 2 min. The survival durations of the mice were monitored, and at 9 weeks after the initial injections, all mice were sacrificed for further histological examination for lung and liver metastasis.

### Construction of lentivirus and stable cell lines

Lentiviral vectors encoding shRNAs were generated using PLKO.1-TRC (Addgene) and designated as LV-shHOXA13, LV-shACLY, LV-shIGF1R, and LV-shcontrol. “LV-shcontrol” is a non-target shRNA control. The vector “pLKO.1-puro Non-Target shRNA Control Plasmid DNA” (purchased from Sigma, SHC016) contains an shRNA insert that does not target any known genes from any species. Short hairpin RNAs (shRNAs) sequences were presented in Supplementary Table S7. Lentiviral vectors encoding the human HOXA13, ACLY, and IGF1R genes were constructed in pLV-puro or pLV-neo (Addgene) and designated as LV-HOXA13, LV-ACLY, and LV-IGF1R. An empty vector was used as the negative control and was designated as LV-control. The lentivirus and cell infection were produced according to the lentiviral vector protocol recommended by Addgene. Briefly, the lentiviral plasmid and packaging plasmids pMD2. G and psPAX2 (Addgene plasmid #12259 and #12260) were transfected into HEK-293T cells with transfection reagent (Lipofectamine®3000, ThermoFisher Scientific) and OPTI-MEM media (Invitrogen, Waltham, MA, USA). The lentiviruses were harvested twice on days 4 and 5. Viruses were filtered with a 0.45-μm filter and stored at −80 °C. Lentiviral infection of target cells were performed in cell culture media with 5 μg/mL polybrene (Sigma H9268). Seventy-two hours after infection, cells were selected for 2 weeks using 2.5 μg/mL puromycin (OriGene). Selected pools of cells were used for the following experiments.

### Agents

Recombinant human IGF1 protein was ordered from Bio-Techne (#291-G1-200, R&D Systems, MN, USA). The PI3K inhibitor LY294002 (#S1105), ERK inhibitor SCH772984 (#S7101), and Linsitinib (OSI-906) were ordered from Selleck Chemicals (Houston, TX, USA). ETC-1002 (HY-12357) was purchased from Med Chem Express (MCE).

### Quantification and statistical analysis

The quantitative data were compared between groups using the Student’s *t*-test. Categorical data were analyzed using Fisher’s exact test. The cumulative recurrence and survival rates were determined using the Kaplan–Meier method and log-rank test. The Cox proportional hazards model was used to determine the independent factors that influence survival and recurrence based on the variables that had been selected from the univariate analyses. A value of *P* < 0.05 was considered to be significant. All the analyses were performed using the SPSS software (version 22.0).

## Supplementary information

Supplementary Figure S1

Supplementary Figure S2

Supplementary Table S1

Supplementary Table S2

Supplementary Table S3

Supplementary Table S4

Supplementary Table S5

Supplementary Table S6

Supplementary Table S7
