# Supplementary figures and images for: IGF1-mediated HOXA13 overexpression promotes colorectal cancer metastasis through upregulating ACLY and IGF1R
Source: Cell Death Dis. 2021 Jun 1;12(6):564. doi: 10.1038/s41419-021-03833-2 (PMC8169856; doi:10.1038/s41419-021-03833-2)

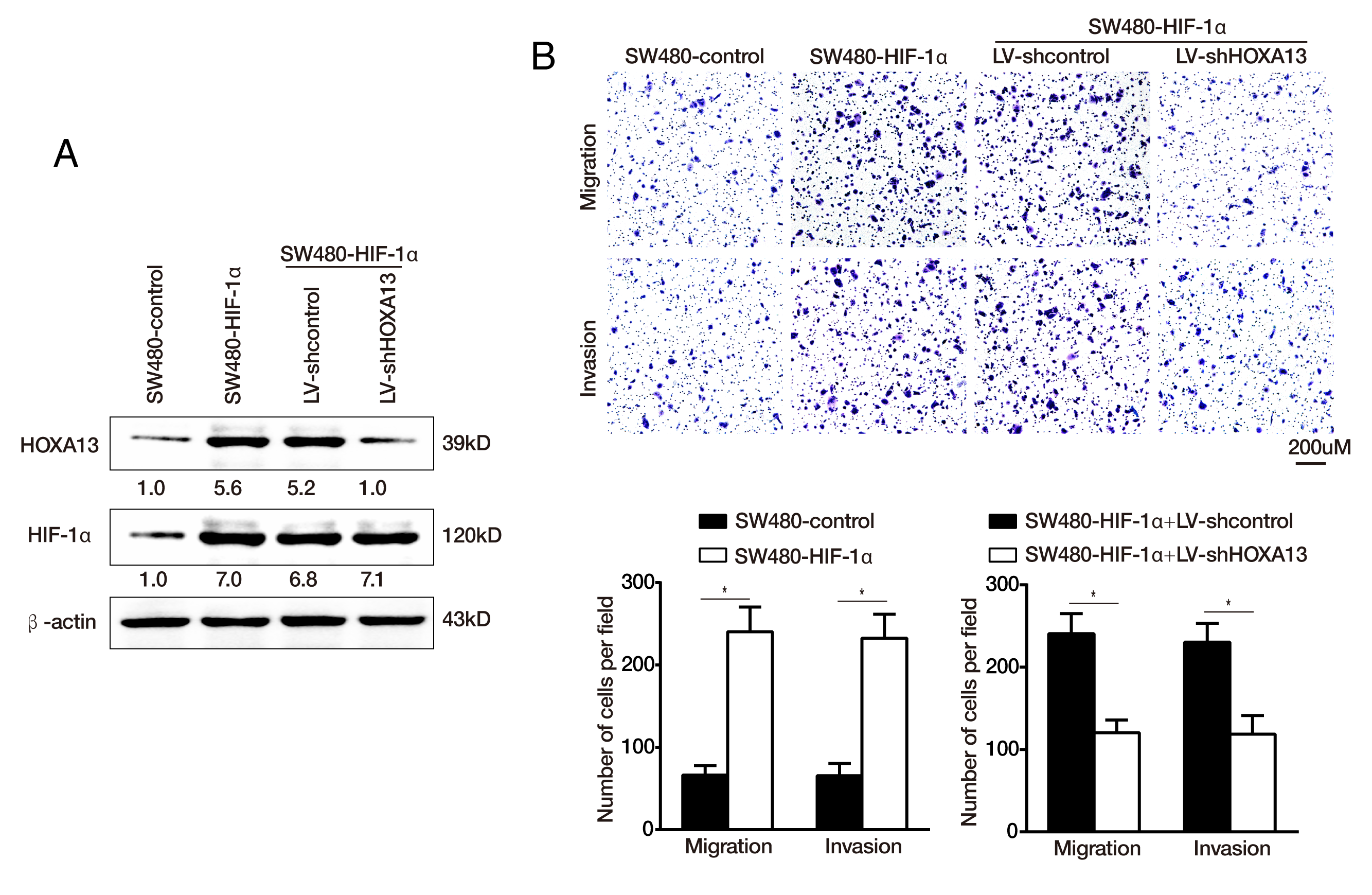

Supplement: Supplementary file 1 — Supplementary Figure S1 [file 41419_2021_3833_MOESM1_ESM.tif]

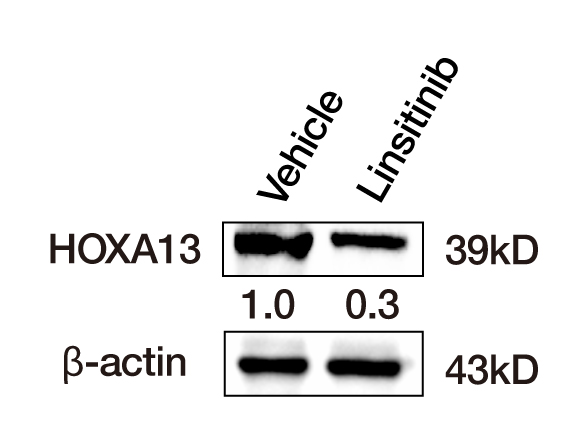

Supplement: Supplementary file 2 — Supplementary Figure S2 [file 41419_2021_3833_MOESM2_ESM.jpg]
