# Supplementary material for: IGF1-mediated HOXA13 overexpression promotes colorectal cancer metastasis through upregulating ACLY and IGF1R
Source: Cell Death Dis. 2021 Jun 1;12(6):564. doi: 10.1038/s41419-021-03833-2 (PMC8169856; doi:10.1038/s41419-021-03833-2)
Supplement: Supplementary file 3 — Supplementary Table S1 [file 41419_2021_3833_MOESM3_ESM.docx]

Supplementary Table S1. Primer sequences used in the study

| **Primer name** | **Primer sequences** | **Enzyme** |
| --- | --- | --- |
| Primers for real-time PCR: |  |  |
| HOXA13 sense | 5’- CTGCCCTATGGCTACTTCGG -3’ |  |
| HOXA13 antisense | 5’- CCGGCGGTATCCATGTACT -3’ |  |
| ACLY sense: | 5’- ATCGGTTCAAGTATGCTCGGG -3’ |  |
| ACLY antisense: | 5’- GACCAAGTTTTCCACGACGTT -3’ |  |
| IGF1R sense | 5'- AGGATATTGGGCTTTACAACCTG -3' |  |
| IGF1R antisense | 5'- GAGGTAACAGAGGTCAGCATTTT -3' |  |
| GAPDH sense: | 5’-CTGGGCTACACTGAGCACC-3’ |  |
| GAPDH antisense: | 5’-AAGTGGTCGTTGAGGGCAATG-3’ |  |
| **Primers for ACLY promoter construct:** |  |  |
| (-1910/+109) ACLY sense: | 5'- TATAGCTAGCTTCCTCTGCCACTTGCTA -3' | Nhel |
| (-1424/+109) ACLY sense: | 5'- TATAGCTAGCAGACAGAGCAAGACTACG -3' | Nhel |
| (-559/+109) ACLY sense: | 5'- TATAGCTAGCAACACAGTGAAACCCCCA -3' | Nhel |
| (-271/+109) ACLY sense: | 5'- TATAGCTAGCAGAAAATTCCCCGCACAG -3' | Nhel |
| antisense: | 5'- ATATCTCGAGTTTTCAGGCAGCAACTCC -3' | XhoI |
| **Primers for ACLY promoter site-directed mutagenesis:** | |  |
| HOXA13 binding site 3 mutation sense: | 5'- CCCGTCTCTAagccAAATACAAAA -3' |  |
| HOXA13 binding site 3mutation antisense: | 5'- TTTTGTATTTggctTAGAGACGGG -3' |  |
| HOXA13 binding site 2 mutation sense: | 5'- GTTATCTGCAcgacAAAGGTGAGG -3' |  |
| HOXA13 binding site 2 mutation antisense: | 5'- CCTCACCTTTgtcgTGCAGATAAC -3' |  |
| HOXA13 binding site 1 mutation sense: | 5'- CCCATCTCTAaccgAAAGACAAAA -3' |  |
| HOXA13 binding site 1 mutation antisense: | 5'- TTTTGTCTTTcggtTAGAGATGGG -3' |  |
| **Primers used for ChIP in the ACLY promoter:** | |  |
| distant region sense: | 5’-GGCTGGTCTCAAACAC-3’ |  |
| distant region antisense: | 5’-ATGCCATTGCACTCTA-3’ |  |
| binding site 3 sense: | 5’-TGGTTCACGCCTGTAA-3’ |  |
| binding site 3 antisense: | 5’-CCATGCTCGGCTAATT-3’ |  |
| binding site 2/1 sense: | 5’-GTCCTCCAGGCATCAT-3’ |  |
| binding site 2/1 antisense: | 5’-GGAGTTTCGCTCTTGTT-3’ |  |
| **Primers for IGF1R promoter construct:** | |  |
| (-2116/+286) IGF1R sense: | 5’-TATAGCTAGCTCTGCAGGAATCAAGACCA-3’ | NheI |
| (-1921/+286) IGF1R sense: | 5’-TATAGCTAGCAGTTAAACAAAACCATCCCA-3’ | NheI |
| (-1720/+286) IGF1R sense: | 5’-TATAGCTAGCACGATGGATACACGTT-3’ | NheI |
| (-496/+286) IGF1R sense: | 5’-TATAGCTAGCTTTTCAAGAACCGGGGAA-3’ | NheI |
| Antisense: | 5’-ATATAGATCTCTCGCTGAAGGTCACA-3’ | BglⅡ |
| **Primers for IGF1R promoter site-directed mutagenesis:** | |  |
| HOXA13 binding site 3 mutation sense: | 5’-TAAGATTCTAcggcATTTATTTCA-3’ |  |
| HOXA13 binding site 3 mutation antisense: | 5’-TGAAATAAATgccgTAGAATCTTA-3’ |  |
| HOXA13 binding site 2 mutation sense: | 5’-TTTTCAGCTCcggcAAAATTATTT-3’ |  |
| HOXA13 binding site 2 mutation antisense: | 5’-AAATAATTTTgccgGAGCTGAAAA-3’ |  |
| HOXA13 binding site 1 mutation sense: | 5’-ATTAGCTATCcggcAAAAATTTAT-3’ |  |
| HOXA13 binding site 1 mutation antisense: | 5’-ATAAATTTTTgccgGATAGCTAAT-3’ |  |
| **Primers used for ChIP in the IGF1R promoter:** | |  |
| distant region sense: | 5’-CTATTATGAACAAAGCAC-3’ |  |
| distant region antisense: | 5’-TTACGATCCAGCAAGT-3’ |  |
| binding site 1 sense: | 5’-CTTCTACCATCCTACCCG-3’ |  |
| binding site 1 antisense: | 5’-TCAAAGAGGGAAACAGC-3’ |  |
| **Primers for HOXA13 promoter construct:** | |  |
| (-1973/+130) HOXA13 sense: | 5’-TATAGCTAGCGGGCCTAGAACCCTACTCT-3’ | Nhel |
| (-1653/+130) HOXA13 sense: | 5’-TATAGCTAGCGCCCAGCCACATTGGTCT-3’ | Nhel |
| (-1019/+130) HOXA13 sense: | 5’-TATAGCTAGCAGTCCCAGCCAGAAGCAC-3’ | Nhel |
| (-166/+130) HOXA13 sense: | 5’-TATAGCTAGCCGCTTTGCATACGCCGTG-3’ | Nhel |
| antisense: | 5’-ATATCTCGAGGCCGCCCCTTCCATGTTC-3’ | XhoI |
| **Primers for HOXA13 promoter site-directed mutagenesis:** | |  |
| FOXO3 binding site mutation sense: | 5’-GTCTAAATGCcggaATATGAAATT-3’ |  |
| FOXO3 binding site mutation antisense: | 5’-AATTTCATATtccgGCATTTAGAC-3’ |  |
| HIF1α binding site mutation sense: | 5’-TGCAGAAATAattaCCGGTTCCTG -3’ |  |
| HIF1α binding site mutation antisense: | 5’- CAGGAACCGGtaatTATTTCTGCA -3’ |  |
| HIF1α binding site mutation sense: | 5’-CCCACCCGCAaacgCCTCCGCCTC-3’ |  |
| HIF1α binding site mutation antisense: | 5’-GAGGCGGAGGcgttTGCGGGTGGG-3’ |  |
| **Primers used for ChIP in the HOXA13 promoter:** | |  |
| distant region sense: | 5’-AACAGAGTCAGAGGCGAGAA-3’ |  |
| distant region antisense: | 5’-CTGCCATGCGAGCGTA-3’ |  |
| binding site 2/1 sense: | 5’-TCCAGATCGCAACCCA-3’ |  |
| binding site 2/1 antisense: | 5’-ACGGCGTATGCAAAGC-3’ |  |
| **Primers for pCMV-HOXA13 construction:** |  |  |
| Sense:5`-TGTTGAATTCATGACAGCCTCCGTGCTCCTC-3` |  | EcoRI |
| Antisense:5`-TTGTCTCGAGTTAACTAGTGGTTTTCAGTT-3` |  | XhoI |
