# Supplementary material for: IGF1-mediated HOXA13 overexpression promotes colorectal cancer metastasis through upregulating ACLY and IGF1R
Source: Cell Death Dis. 2021 Jun 1;12(6):564. doi: 10.1038/s41419-021-03833-2 (PMC8169856; doi:10.1038/s41419-021-03833-2)
Supplement: Supplementary file 4 — Supplementary Table S2 [file 41419_2021_3833_MOESM4_ESM.docx]

Supplementary Table S2. List of genes differentially expressed in SW480-HOXA13 versus SW480-control cells using human Cell Motility and Cancer PathwayFinder PCR array.

| \| **Symbol** \| **SW480-HOXA13**  **vs.**  **SW480-control** \| **Description** \| \| --- \| --- \| --- \|   **Cell Motility PCR array:** | | |  | Description |
| --- | --- | --- | --- | --- | --- | --- | --- |
| \| **IGF1R** \| 6.52 \| Insulin-like growth factor I receptor \| \| --- \| --- \| --- \| \| ACTR2 \| 5.07 \| ARP2 actin-related protein 2 homolog (yeast) \| \| AKT1 \| 4.82 \| Thymoma viral proto-oncogene 1 \| \| CDC42 \| 3.94 \| Cell division cycle 42 homolog (S. cerevisiae) \| \| ITGB1 \| 3.91 \| Integrin beta 1 (fibronectin receptor beta) \| \| MMP9 \| 3.86 \| Matrix metallopeptidase 9 \| \| TGFB1 \| 3.86 \| Transforming growth factor, beta 1 \| \| EGFR \| 3.82 \| Epidermal growth factor receptor \| \| STAT3 \| 3.77 \| Signal transducer and activator of transcription 3 \| \| IGF1 \| 3.63 \| Insulin-like growth factor 1 \| \| ACTN4 \| 3.46 \| Actinin alpha 4 \| \| VEGFA \| 2.93 \| Vascular endothelial growth factor A \| \| VIM \| 2.83 \| Vimentin \| \| ITGA4 \| 2.68 \| Integrin alpha 4 \| \| CAPN1 \| 2.45 \| Calpain 1 \| \| VASP \| 2.36 \| Vasodilator-stimulated phosphoprotein \| \| FGF2 \| 2.28 \| Fibroblast growth factor 2 \| \| ACTN1 \| 2.23 \| Actinin, alpha 1 \| \| BCAR1 \| 2.04 \| Breast cancer anti-estrogen resistance 1 \| \| MET \| 2.04 \| Met proto-oncogene \| \| HGF \| 1.95 \| Hepatocyte growth factor \| \| EGF \| 1.93 \| Epidermal growth factor \| \| ACTN3 \| 1.91 \| Actinin alpha 3 \| \| MMP2 \| 1.91 \| Matrix metallopeptidase 2 \| \| DPP4 \| 1.89 \| Dipeptidylpeptidase 4 \| \| ILK \| 1.87 \| Integrin linked kinase \| \| ACTR3 \| 1.86 \| ARP3 actin-related protein 3 homolog (yeast) \| \| CAPN2 \| 1.85 \| Calpain 2 \| \| PAK1 \| 1.84 \| P21 protein (Cdc42/Rac)-activated kinase 1 \| \| ARF6 \| 1.82 \| ADP-ribosylation factor 6 \| \| ITGB3 \| 1.79 \| Integrin beta 3 \| \| MAPK1 \| 1.76 \| Mitogen-activated protein kinase 1 \| \| CSF1 \| 1.76 \| Colony stimulating factor 1 (macrophage) \| \| MMP14 \| 1.76 \| Matrix metallopeptidase 14 (membrane-inserted) \| \| CTTN \| 1.68 \| Cortactin \| \| EZR \| 1.59 \| Ezrin \| \| PLD1 \| 1.55 \| Phospholipase D1 \| \| ARHGEF7 \| 1.52 \| Rho guanine nucleotide exchange factor (GEF7) \| \| RAC1 \| 1.51 \| RAS-related C3 botulinum substrate 1 \| \| FAP \| 1.49 \| Fibroblast activation protein \| \| SRC \| 1.48 \| Rous sarcoma oncogene \| \| ARHGDIA \| 1.48 \| Rho GDP dissociation inhibitor (GDI) alpha \| \| RASA1 \| 1.44 \| RAS p21 protein activator 1 \| \| CFL1 \| 1.41 \| Cofilin 1, non-muscle \| \| ENAH \| 1.35 \| Enabled homolog (Drosophila) \| \| BAIAP2 \| 1.33 \| Brain-specific angiogenesis inhibitor 1-associated protein 2 \| \| ROCK1 \| 1.33 \| Rho-associated coiled-coil containing protein kinase 1 \| \| ITGB2 \| 1.28 \| Integrin beta 2 \| \| PTK2 \| 1.25 \| PTK2 protein tyrosine kinase 2 \| \| MYH9 \| 1.23 \| Myosin, heavy polypeptide 9, non-muscle \| \| CAV1 \| 1.16 \| Caveolin 1, caveolae protein \| \| PAK4 \| 1.15 \| P21 protein (Cdc42/Rac)-activated kinase 4 \| \| LIMK1 \| 1.13 \| LIM-domain containing, protein kinase \| \| RAC2 \| 1.08 \| RAS-related C3 botulinum substrate 2 \| \| TLN1 \| 1.05 \| Talin 1 \| \| PTK2B \| 1.05 \| PTK2 protein tyrosine kinase 2 beta \| \| PRKCA \| 1.03 \| Protein kinase C, alpha \| \| MYLK \| 1.02 \| Myosin, light polypeptide kinase \| \| RHOC \| 1.01 \| Ras homolog gene family, member C \| \| PLAUR \| -1.01 \| Plasminogen activator, urokinase receptor \| \| RHOA \| -1.01 \| Ras homolog gene family, member A \| \| SVIL \| -1.02 \| Supervillin \| \| RND3 \| -1.02 \| Rho family GTPase 3 \| \| WASL \| -1.03 \| Wiskott-Aldrich syndrome-like (human) \| \| PTEN \| -1.08 \| Phosphatase and tensin homolog \| \| PXN \| -1.12 \| Paxillin \| \| MYH10 \| -1.15 \| Myosin, heavy polypeptide 10, non-muscle \| \| WASF2 \| -1.16 \| WAS protein family, member 2 \| \| PIK3CA \| -1.22 \| Phosphatidylinositol 3-kinase, catalytic, alpha polypeptide \| \| MSN \| -1.23 \| Moesin \| \| RHOB \| -1.25 \| Ras homolog gene family, member B \| \| VCL \| -1.26 \| Vinculin \| \| 2900073G15 RIK \| -1.28 \| RIKEN cDNA 2900073G15 gene \| \| DIAP1 \| -1.29 \| Diaphanous homolog 1 (Drosophila) \| \| CRK \| -1.29 \| V-crk sarcoma virus CT10 oncogene homolog (avian) \| \| TIMP2 \| -1.33 \| Tissue inhibitor of metalloproteinase 2 \| \| PTPN1 \| -1.44 \| Protein tyrosine phosphatase, non-receptor type 1 \| \| SH3PXD2A \| -1.49 \| SH3 and PX domains 2A \| \| WASF1 \| -1.53 \| WASP family 1 \| \| RHO \| -1.56 \| Rhodopsin \| \| WIPF1 \| -1.67 \| WAS/WASL interacting protein family, member 1 \| \| PFN1 \| -1.74 \| Profilin 1 \| \| PLCG1 \| -1.82 \| Phospholipase C, gamma 1 \| \| RDX \| -1.95 \| Radixin \|   **Cancer PathwayFinder PCR array:**   \| **ACLY** \| 5.46 \| ATP citrate lyase \| \| --- \| --- \| --- \| \| SOD1 \| 4.28 \| Superoxide dismutase 1, soluble \| \| CCL2 \| 4.13 \| Chemokine (C-C motif) ligand 2 \| \| CDH2 \| 3.85 \| Cadherin 2, type 1, N-cadherin (neuronal) \| \| E2F4 \| 3.66 \| E2F transcription factor 4, p107/p130-binding \| \| FLT4 \| 3.54 \| Fms-related tyrosine kinase 4 (vascular endothelial growth factor/vascular permeability factor receptor) \| \| ACSL4 \| 3.27 \| Acyl-CoA synthetase long-chain family member 4 \| \| G6PD \| 3.08 \| Glucose-6-phosphate dehydrogenase \| \| VEGFA \| 2.83 \| Vascular endothelial growth factor A \| \| BIRC3 \| 2.81 \| Baculoviral IAP repeat containing 3 \| \| XIAP \| 2.81 \| X-linked inhibitor of apoptosis \| \| BMI1 \| 2.77 \| BMI1 polycomb ring finger oncogene \| \| IGFBP3 \| 2.76 \| Insulin-like growth factor binding protein 3 \| \| FGF2 \| 2.56 \| Fibroblast growth factor 2 (basic) \| \| IGFBP5 \| 2.34 \| Insulin-like growth factor binding protein 5 \| \| ANGPT2 \| 2.17 \| Angiopoietin 2 \| \| ADM \| 2.11 \| Adrenomedullin \| \| LDHA \| 2.09 \| Lactate dehydrogenase A \| \| LPL \| 2.08 \| Lipoprotein lipase \| \| MCM2 \| 2.04 \| Minichromosome maintenance complex component 2 \| \| DSP \| 1.89 \| Desmoplakin \| \| ETS2 \| 1.84 \| V-Ets erythroblastosis virus E26 oncogene homolog 2 (avian) \| \| ANGPT1 \| 1.79 \| Angiopoietin 1 \| \| CFLAR \| 1.78 \| CASP8 and FADD-like apoptosis regulator \| \| COX5A \| 1.78 \| Cytochrome c oxidase subunit Va \| \| SNAI1 \| 1.76 \| Snail homolog 1 (Drosophila) \| \| ATP5A1 \| 1.76 \| ATP synthase, H+ transporting, mitochondrial F1 complex, alpha subunit 1, cardiac muscle \| \| DDB2 \| 1.74 \| Damage-specific DNA binding protein 2, 48kDa \| \| ARNT \| 1.74 \| Aryl hydrocarbon receptor nuclear translocator \| \| CDC20 \| 1.71 \| Cell division cycle 20 homolog (S. cerevisiae) \| \| AURKA \| 1.68 \| Aurora kinase A \| \| EPO \| 1.66 \| Erythropoietin \| \| SOX10 \| 1.65 \| SRY (sex determining region Y)-box 10 \| \| DKC1 \| 1.63 \| Dyskeratosis congenita 1, dyskerin \| \| ERCC3 \| 1.61 \| Excision repair cross-complementing rodent repair deficiency, complementation group 3 (xeroderma pigmentosum group B complementing) \| \| CA9 \| 1.59 \| Carbonic anhydrase IX \| \| MAP2K1 \| 1.59 \| Mitogen-activated protein kinase kinase 1 \| \| SNAI2 \| 1.58 \| Snail homolog 2 (Drosophila) \| \| FASLG \| 1.56 \| Fas ligand (TNF superfamily, member 6) \| \| GSC \| 1.56 \| Goosecoid homeobox \| \| KDR \| 1.55 \| Kinase insert domain receptor (a type III receptor tyrosine kinase) \| \| STMN1 \| 1.51 \| Stathmin 1 \| \| LIG4 \| 1.51 \| Ligase IV, DNA, ATP-dependent \| \| TBX2 \| 1.51 \| T-box 2 \| \| TEK \| 1.49 \| TEK tyrosine kinase, endothelial \| \| MKI67 \| 1.48 \| Antigen identified by monoclonal antibody Ki-67 \| \| NOL3 \| 1.46 \| Nucleolar protein 3 (apoptosis repressor with CARD domain) \| \| PFKL \| 1.46 \| Phosphofructokinase, liver \| \| SNAI3 \| 1.45 \| Snail homolog 3 (Drosophila) \| \| POLB \| 1.45 \| Polymerase (DNA directed), beta \| \| WEE1 \| 1.44 \| WEE1 homolog (S. pombe) \| \| UQCRFS1 \| 1.38 \| Ubiquinol-cytochrome c reductase, Rieske iron-sulfur polypeptide 1 \| \| FOXC2 \| 1.37 \| Forkhead box C2 (MFH-1, mesenchyme forkhead 1) \| \| TINF2 \| 1.36 \| TERF1 (TRF1)-interacting nuclear factor 2 \| \| OCLN \| 1.33 \| Occludin \| \| TERF1 \| 1.33 \| Telomeric repeat binding factor (NIMA-interacting) 1 \| \| PGF \| 1.32 \| Placental growth factor \| \| SLC2A1 \| 1.28 \| Solute carrier family 2 (facilitated glucose transporter), member 1 \| \| SERPINB2 \| 1.21 \| Serpin peptidase inhibitor, clade B (ovalbumin), member 2 \| \| TNKS \| 1.04 \| Tankyrase, TRF1-interacting ankyrin-related ADP-ribose polymerase \| \| SKP2 \| 1.02 \| S-phase kinase-associated protein 2 (p45) \| \| KRT14 \| 1.01 \| Keratin 14 \| \| GADD45G \| -1.01 \| Growth arrest and DNA-damage-inducible, gamma \| \| BCL2L11 \| -1.03 \| BCL2-like 11 (apoptosis facilitator) \| \| CCND3 \| -1.03 \| Cyclin D3 \| \| TNKS2 \| -1.06 \| Tankyrase, TRF1-interacting ankyrin-related ADP-ribose polymerase 2 \| \| CASP7 \| -1.11 \| Caspase 7, apoptosis-related cysteine peptidase \| \| ERCC5 \| -1.16 \| Excision repair cross-complementing rodent repair deficiency, complementation group 5 \| \| TEP1 \| -1.19 \| Telomerase-associated protein 1 \| \| CPT2 \| -1.26 \| Carnitine palmitoyltransferase 2 \| \| MAPK14 \| -1.34 \| Mitogen-activated protein kinase 14 \| \| PPP1R15A \| -1.42 \| Protein phosphatase 1, regulatory (inhibitor) subunit 15A \| \| IGFBP7 \| -1.48 \| Insulin-like growth factor binding protein 7 \| \| MAP2K3 \| -1.59 \| Mitogen-activated protein kinase kinase 3 \| \| TERF2IP \| -1.71 \| Telomeric repeat binding factor 2, interacting protein \| \| PINX1 \| -1.76 \| PIN2/TERF1 interacting, telomerase inhibitor 1 \| \| CASP9 \| -1.84 \| Caspase 9, apoptosis-related cysteine peptidase \| \| GPD2 \| -1.95 \| Glycerol-3-phosphate dehydrogenase 2 (mitochondrial) \| \| CCND2 \| -2.01 \| Cyclin D2 \| \| SERPINF1 \| -2.14 \| Serpin peptidase inhibitor, clade F (alpha-2 antiplasmin, pigment epithelium derived factor), member 1 \| \| APAF1 \| -2.14 \| Apoptotic peptidase activating factor 1 \| \| DDIT3 \| -2.25 \| DNA-damage-inducible transcript 3 \| \| CASP2 \| -2.27 \| Caspase 2, apoptosis-related cysteine peptidase \| \| HMOX1 \| -2.31 \| Heme oxygenase (decycling) 1 \| | |  | |  |
|  |  | | |  |
