# Supplementary material for: IGF1-mediated HOXA13 overexpression promotes colorectal cancer metastasis through upregulating ACLY and IGF1R
Source: Cell Death Dis. 2021 Jun 1;12(6):564. doi: 10.1038/s41419-021-03833-2 (PMC8169856; doi:10.1038/s41419-021-03833-2)
Supplement: Supplementary file 5 — Supplementary Table S3 [file 41419_2021_3833_MOESM5_ESM.docx]

Supplementary Table S3. List of genes differentially expressed in DLD-1-HOXA13 versus DLD-1-control cells using human Cell Motility and Cancer PathwayFinder PCR array.

| \| **Symbol** \| **DLD-1-HOXA13**  **vs.**  **DLD-1-control** \| **Description** \| \| --- \| --- \| --- \|   **Cell Motility PCR array:** | |  | Description |
| --- | --- | --- | --- | --- | --- | --- |
| \| \| ITGB3 \| 5.64 \| Integrin beta 3 \| \| --- \| --- \| --- \| \| CSF1 \| 4.33 \| Colony stimulating factor 1 (macrophage) \| \| **IGF1R** \| 4.25 \| Insulin-like growth factor I receptor \| \| MYH9 \| 3.59 \| Myosin, heavy polypeptide 9, non-muscle \| \| MMP2 \| 3.51 \| Matrix metallopeptidase 2 \| \| SRC \| 3.44 \| Rous sarcoma oncogene \| \| ITGB2 \| 3.17 \| Integrin beta 2 \| \| CAPN2 \| 2.89 \| Calpain 2 \| \| IGF1 \| 2.83 \| Insulin-like growth factor 1 \| \| MAPK1 \| 2.76 \| Mitogen-activated protein kinase 1 \| \| RAC1 \| 2.55 \| RAS-related C3 botulinum substrate 1 \| \| ARF6 \| 2.54 \| ADP-ribosylation factor 6 \| \| PAK1 \| 2.52 \| P21 protein (Cdc42/Rac)-activated kinase 1 \| \| EGF \| 2.48 \| Epidermal growth factor \| \| RASA1 \| 2.48 \| RAS p21 protein activator 1 \| \| TLN1 \| 2.26 \| Talin 1 \| \| HGF \| 2.14 \| Hepatocyte growth factor \| \| VASP \| 1.96 \| Vasodilator-stimulated phosphoprotein \| \| EGFR \| 1.91 \| Epidermal growth factor receptor \| \| MMP9 \| 1.91 \| Matrix metallopeptidase 9 \| \| ITGA4 \| 1.87 \| Integrin alpha 4 \| \| AKT1 \| 1.82 \| Thymoma viral proto-oncogene 1 \| \| CDC42 \| 1.81 \| Cell division cycle 42 homolog (S. cerevisiae) \| \| ITGB1 \| 1.77 \| Integrin beta 1 (fibronectin receptor beta) \| \| VEGFA \| 1.75 \| Vascular endothelial growth factor A \| \| VIM \| 1.73 \| Vimentin \| \| STAT3 \| 1.72 \| Signal transducer and activator of transcription 3 \| \| TGFB1 \| 1.66 \| Transforming growth factor, beta 1 \| \| RAC2 \| 1.66 \| RAS-related C3 botulinum substrate 2 \| \| ACTN4 \| 1.63 \| Actinin alpha 4 \| \| ENAH \| 1.62 \| Enabled homolog (Drosophila) \| \| FGF2 \| 1.56 \| Fibroblast growth factor 2 \| \| LIMK1 \| 1.53 \| LIM-domain containing, protein kinase \| \| ACTR2 \| 1.48 \| ARP2 actin-related protein 2 homolog (yeast) \| \| ACTN3 \| 1.44 \| Actinin alpha 3 \| \| MET \| 1.44 \| Met proto-oncogene \| \| PAK4 \| 1.41 \| P21 protein (Cdc42/Rac)-activated kinase 4 \| \| MYLK \| 1.39 \| Myosin, light polypeptide kinase \| \| RHOC \| 1.39 \| Ras homolog gene family, member C \| \| PTK2B \| 1.38 \| PTK2 protein tyrosine kinase 2 beta \| \| ILK \| 1.35 \| Integrin linked kinase \| \| ACTN1 \| 1.34 \| Actinin, alpha 1 \| \| BAIAP2 \| 1.33 \| Brain-specific angiogenesis inhibitor 1-associated protein 2 \| \| ACTR3 \| 1.31 \| ARP3 actin-related protein 3 homolog (yeast) \| \| PLAUR \| 1.31 \| Plasminogen activator, urokinase receptor \| \| CAPN1 \| 1.28 \| Calpain 1 \| \| BCAR1 \| 1.28 \| Breast cancer anti-estrogen resistance 1 \| \| CAV1 \| 1.25 \| Caveolin 1, caveolae protein \| \| PLD1 \| 1.24 \| Phospholipase D1 \| \| MMP14 \| 1.24 \| Matrix metallopeptidase 14 (membrane-inserted) \| \| DPP4 \| 1.23 \| Dipeptidylpeptidase 4 \| \| WASL \| 1.22 \| Wiskott-Aldrich syndrome-like (human) \| \| PRKCA \| 1.15 \| Protein kinase C, alpha \| \| ARHGEF7 \| 1.15 \| Rho guanine nucleotide exchange factor (GEF7) \| \| FAP \| 1.12 \| Fibroblast activation protein \| \| CTTN \| 1.11 \| Cortactin \| \| ROCK1 \| 1.09 \| Rho-associated coiled-coil containing protein kinase 1 \| \| ARHGDIA \| 1.08 \| Rho GDP dissociation inhibitor (GDI) alpha \| \| CFL1 \| 1.06 \| Cofilin 1, non-muscle \| \| EZR \| 1.06 \| Ezrin \| \| RDX \| 1.05 \| Radixin \| \| PTK2 \| 1.03 \| PTK2 protein tyrosine kinase 2 \| \| WASF2 \| 1.02 \| WAS protein family, member 2 \| \| PIK3CA \| 1.01 \| Phosphatidylinositol 3-kinase, catalytic, alpha polypeptide \| \| WASF1 \| 1.01 \| WASP family 1 \| \| PTEN \| -1.02 \| Phosphatase and tensin homolog \| \| 2900073G15 RIK \| -1.01 \| RIKEN cDNA 2900073G15 gene \| \| DIAP1 \| -1.01 \| Diaphanous homolog 1 (Drosophila) \| \| CRK \| -1.01 \| V-crk sarcoma virus CT10 oncogene homolog (avian) \| \| RHOB \| -1.01 \| Ras homolog gene family, member B \| \| PXN \| -1.01 \| Paxillin \| \| RHOA \| -1.02 \| Ras homolog gene family, member A \| \| SVIL \| -1.05 \| Supervillin \| \| RND3 \| -1.14 \| Rho family GTPase 3 \| \| PLCG1 \| -1.16 \| Phospholipase C, gamma 1 \| \| MSN \| -1.22 \| Moesin \| \| MYH10 \| -1.23 \| Myosin, heavy polypeptide 10, non-muscle \| \| WIPF1 \| -1.26 \| WAS/WASL interacting protein family, member 1 \| \| SH3PXD2A \| -1.26 \| SH3 and PX domains 2A \| \| RHO \| -1.27 \| Rhodopsin \| \| PTPN1 \| -1.29 \| Protein tyrosine phosphatase, non-receptor type 1 \| \| TIMP2 \| -1.29 \| Tissue inhibitor of metalloproteinase 2 \| \| VCL \| -1.31 \| Vinculin \| \| PFN1 \| -1.38 \| Profilin 1 \| \|  \|  \| \| --- \| --- \| --- \| --- \| --- \| --- \| --- \| --- \| --- \| --- \| --- \| --- \| --- \| --- \| --- \| --- \| --- \| --- \| --- \| --- \| --- \| --- \| --- \| --- \| --- \| --- \| --- \| --- \| --- \| --- \| --- \| --- \| --- \| --- \| --- \| --- \| --- \| --- \| --- \| --- \| --- \| --- \| --- \| --- \| --- \| --- \| --- \| --- \| --- \| --- \| --- \| --- \| --- \| --- \| --- \| --- \| --- \| --- \| --- \| --- \| --- \| --- \| --- \| --- \| --- \| --- \| --- \| --- \| --- \| --- \| --- \| --- \| --- \| --- \| --- \| --- \| --- \| --- \| --- \| --- \| --- \| --- \| --- \| --- \| --- \| --- \| --- \| --- \| --- \| --- \| --- \| --- \| --- \| --- \| --- \| --- \| --- \| --- \| --- \| --- \| --- \| --- \| --- \| --- \| --- \| --- \| --- \| --- \| --- \| --- \| --- \| --- \| --- \| --- \| --- \| --- \| --- \| --- \| --- \| --- \| --- \| --- \| --- \| --- \| --- \| --- \| --- \| --- \| --- \| --- \| --- \| --- \| --- \| --- \| --- \| --- \| --- \| --- \| --- \| --- \| --- \| --- \| --- \| --- \| --- \| --- \| --- \| --- \| --- \| --- \| --- \| --- \| --- \| --- \| --- \| --- \| --- \| --- \| --- \| --- \| --- \| --- \| --- \| --- \| --- \| --- \| --- \| --- \| --- \| --- \| --- \| --- \| --- \| --- \| --- \| --- \| --- \| --- \| --- \| --- \| --- \| --- \| --- \| --- \| --- \| --- \| --- \| --- \| --- \| --- \| --- \| --- \| --- \| --- \| --- \| --- \| --- \| --- \| --- \| --- \| --- \| --- \| --- \| --- \| --- \| --- \| --- \| --- \| --- \| --- \| --- \| --- \| --- \| --- \| --- \| --- \| --- \| --- \| --- \| --- \| --- \| --- \| --- \| --- \| --- \| --- \| --- \| --- \| --- \| --- \| --- \| --- \| --- \| --- \| --- \| --- \| --- \| --- \| --- \| --- \| --- \| --- \| --- \| --- \| --- \| --- \| --- \| --- \| --- \| --- \| --- \| --- \| --- \| --- \| --- \| \|  \|  \|  \|   **Cancer PathwayFinder PCR array:**   \| ACSL4 \| 5.22 \| Acyl-CoA synthetase long-chain family member 4 \| \| --- \| --- \| --- \| \| IGFBP3 \| 5.18 \| Insulin-like growth factor binding protein 3 \| \| **ACLY** \| 5.11 \| ATP citrate lyase \| \| ATP5A1 \| 4.65 \| ATP synthase, H+ transporting, mitochondrial F1 complex, alpha subunit 1, cardiac muscle \| \| FASLG \| 4.33 \| Fas ligand (TNF superfamily, member 6) \| \| ARNT \| 4.25 \| Aryl hydrocarbon receptor nuclear translocator \| \| AURKA \| 4.25 \| Aurora kinase A \| \| SNAI2 \| 4.11 \| Snail homolog 2 (Drosophila) \| \| CA9 \| 4.02 \| Carbonic anhydrase IX \| \| FOXC2 \| 3.87 \| Forkhead box C2 (MFH-1, mesenchyme forkhead 1) \| \| CDC20 \| 3.56 \| Cell division cycle 20 homolog (S. cerevisiae) \| \| ETS2 \| 3.45 \| V-Ets erythroblastosis virus E26 oncogene homolog 2 (avian) \| \| ANGPT1 \| 3.28 \| Angiopoietin 1 \| \| CFLAR \| 3.07 \| CASP8 and FADD-like apoptosis regulator \| \| COX5A \| 2.79 \| Cytochrome c oxidase subunit Va \| \| SLC2A1 \| 2.74 \| Solute carrier family 2 (facilitated glucose transporter), member 1 \| \| PGF \| 2.56 \| Placental growth factor \| \| DDB2 \| 2.34 \| Damage-specific DNA binding protein 2, 48kDa \| \| E2F4 \| 1.99 \| E2F transcription factor 4, p107/p130-binding \| \| DKC1 \| 1.98 \| Dyskeratosis congenita 1, dyskerin \| \| DSP \| 1.98 \| Desmoplakin \| \| EPO \| 1.97 \| Erythropoietin \| \| BIRC3 \| 1.95 \| Baculoviral IAP repeat containing 3 \| \| FGF2 \| 1.91 \| Fibroblast growth factor 2 (basic) \| \| ERCC3 \| 1.88 \| Excision repair cross-complementing rodent repair deficiency, complementation group 3 (xeroderma pigmentosum group B complementing) \| \| FLT4 \| 1.87 \| Fms-related tyrosine kinase 4 (vascular endothelial growth factor/vascular permeability factor receptor) \| \| XIAP \| 1.86 \| X-linked inhibitor of apoptosis \| \| ADM \| 1.84 \| Adrenomedullin \| \| SNAI1 \| 1.82 \| Snail homolog 1 (Drosophila) \| \| BMI1 \| 1.78 \| BMI1 polycomb ring finger oncogene \| \| G6PD \| 1.78 \| Glucose-6-phosphate dehydrogenase \| \| IGFBP5 \| 1.78 \| Insulin-like growth factor binding protein 5 \| \| CCL2 \| 1.77 \| Chemokine (C-C motif) ligand 2 \| \| VEGFA \| 1.76 \| Vascular endothelial growth factor A \| \| GSC \| 1.76 \| Goosecoid homeobox \| \| KDR \| 1.74 \| Kinase insert domain receptor (a type III receptor tyrosine kinase) \| \| STMN1 \| 1.72 \| Stathmin 1 \| \| LIG4 \| 1.71 \| Ligase IV, DNA, ATP-dependent \| \| SOX10 \| 1.71 \| SRY (sex determining region Y)-box 10 \| \| LPL \| 1.69 \| Lipoprotein lipase \| \| MAP2K1 \| 1.69 \| Mitogen-activated protein kinase kinase 1 \| \| MCM2 \| 1.67 \| Minichromosome maintenance complex component 2 \| \| SNAI3 \| 1.64 \| Snail homolog 3 (Drosophila) \| \| TINF2 \| 1.64 \| TERF1 (TRF1)-interacting nuclear factor 2 \| \| MKI67 \| 1.63 \| Antigen identified by monoclonal antibody Ki-67 \| \| NOL3 \| 1.63 \| Nucleolar protein 3 (apoptosis repressor with CARD domain) \| \| OCLN \| 1.58 \| Occludin \| \| PFKL \| 1.56 \| Phosphofructokinase, liver \| \| CDH2 \| 1.55 \| Cadherin 2, type 1, N-cadherin (neuronal) \| \| TERF1 \| 1.53 \| Telomeric repeat binding factor (NIMA-interacting) 1 \| \| LDHA \| 1.48 \| Lactate dehydrogenase A \| \| SERPINB2 \| 1.48 \| Serpin peptidase inhibitor, clade B (ovalbumin), member 2 \| \| SOD1 \| 1.48 \| Superoxide dismutase 1, soluble \| \| ANGPT2 \| 1.46 \| Angiopoietin 2 \| \| TNKS \| 1.44 \| Tankyrase, TRF1-interacting ankyrin-related ADP-ribose polymerase \| \| SKP2 \| 1.39 \| S-phase kinase-associated protein 2 (p45) \| \| TBX2 \| 1.38 \| T-box 2 \| \| TEK \| 1.33 \| TEK tyrosine kinase, endothelial \| \| UQCRFS1 \| 1.26 \| Ubiquinol-cytochrome c reductase, Rieske iron-sulfur polypeptide 1 \| \| KRT14 \| 1.16 \| Keratin 14 \| \| POLB \| 1.07 \| Polymerase (DNA directed), beta \| \| WEE1 \| 1.01 \| WEE1 homolog (S. pombe) \| \| MAPK14 \| -1.01 \| Mitogen-activated protein kinase 14 \| \| CASP2 \| -1.01 \| Caspase 2, apoptosis-related cysteine peptidase \| \| BCL2L11 \| -1.03 \| BCL2-like 11 (apoptosis facilitator) \| \| HMOX1 \| -1.03 \| Heme oxygenase (decycling) 1 \| \| CASP9 \| -1.14 \| Caspase 9, apoptosis-related cysteine peptidase \| \| APAF1 \| -1.18 \| Apoptotic peptidase activating factor 1 \| \| CASP7 \| -1.23 \| Caspase 7, apoptosis-related cysteine peptidase \| \| GPD2 \| -1.29 \| Glycerol-3-phosphate dehydrogenase 2 (mitochondrial) \| \| TEP1 \| -1.33 \| Telomerase-associated protein 1 \| \| CPT2 \| -1.37 \| Carnitine palmitoyltransferase 2 \| \| ERCC5 \| -1.42 \| Excision repair cross-complementing rodent repair deficiency, complementation group 5 \| \| GADD45G \| -1.49 \| Growth arrest and DNA-damage-inducible, gamma \| \| MAP2K3 \| -1.56 \| Mitogen-activated protein kinase kinase 3 \| \| TERF2IP \| -1.63 \| Telomeric repeat binding factor 2, interacting protein \| \| CCND3 \| -1.63 \| Cyclin D3 \| \| PINX1 \| -1.72 \| PIN2/TERF1 interacting, telomerase inhibitor 1 \| \| PPP1R15A \| -1.77 \| Protein phosphatase 1, regulatory (inhibitor) subunit 15A \| \| CCND2 \| -1.79 \| Cyclin D2 \| \| SERPINF1 \| -1.86 \| Serpin peptidase inhibitor, clade F (alpha-2 antiplasmin, pigment epithelium derived factor), member 1 \| \| IGFBP7 \| -1.92 \| Insulin-like growth factor binding protein 7 \| \| DDIT3 \| -1.98 \| DNA-damage-inducible transcript 3 \| \| TNKS2 \| -2.03 \| Tankyrase, TRF1-interacting ankyrin-related ADP-ribose polymerase 2 \| \|  \|  \|  \| |  | |  |
