# Supplementary material for: IGF1-mediated HOXA13 overexpression promotes colorectal cancer metastasis through upregulating ACLY and IGF1R
Source: Cell Death Dis. 2021 Jun 1;12(6):564. doi: 10.1038/s41419-021-03833-2 (PMC8169856; doi:10.1038/s41419-021-03833-2)
Supplement: Supplementary file 6 — Supplementary Table S4 [file 41419_2021_3833_MOESM6_ESM.docx]

Supplementary Table S4. Correlation between IGF1 expression and clinicopathological characteristics of CRCs in two independent cohorts of human CRC tissues

|  |  | Cohort I (n=342) | |  |  | Cohort II (n=377) | |  |
| --- | --- | --- | --- | --- | --- | --- | --- | --- |
| Clinicopathological variables | | Tumor IGF1 expression | | p Value |  | Tumor IGF1 expression | | p Value |
|  |  | Negative  (n=202) | Positive (n=140) |  |  | Negative  (n=222) | Positive  (n=155) |  |
| Age | | 66.35(11.60) | 66.02(10.89) | 0.373 |  | 67.52(11.77) | 67.47(11.39) | 0.966 |
| Sex | female | 82 | 68 | 0.151 |  | 92 | 77 | 0.114 |
|  | male | 120 | 72 |  |  | 130 | 78 |  |
| Tumor location | right colon | 90 | 60 | 0.759 |  | 89 | 71 | 0508 |
|  | left colon | 85 | 64 |  |  | 100 | 65 |  |
|  | rectum | 27 | 16 |  |  | 33 | 19 |  |
| Tumor size | ＜5cm | 81 | 63 | 0.375 |  | 81 | 62 | 0.489 |
|  | ≥5cm | 121 | 77 |  |  | 141 | 93 |  |
| Tumor differentiation | well or moderate | 163 | 70 | <0.001 |  | 140 | 72 | 0.001 |
|  | poor | 39 | 70 |  |  | 82 | 83 |  |
| Tumor invasion | T1 | 5 | 2 | 0.012 |  | 13 | 2 | 0.048 |
|  | T2 | 25 | 5 |  |  | 15 | 9 |  |
|  | T3 | 132 | 92 |  |  | 156 | 105 |  |
|  | T4 | 40 | 41 |  |  | 38 | 39 |  |
| Lymph node metastasis | absent | 142 | 43 | <0.001 |  | 161 | 52 | <0.001 |
|  | present | 60 | 97 |  |  | 61 | 103 |  |
| Distant metastasis | absent | 184 | 93 | <0.001 |  | 195 | 110 | <0.001 |
|  | present | 18 | 47 |  |  | 27 | 45 |  |
| AJCC stage | Stage I | 30 | 5 | <0.001 |  | 15 | 4 | <0.001 |
|  | Stage II | 111 | 34 |  |  | 143 | 44 |  |
|  | Stage III | 43 | 54 |  |  | 38 | 63 |  |
|  | Stage IV | 18 | 47 |  |  | 26 | 44 |  |
