# Supplementary material for: IGF1-mediated HOXA13 overexpression promotes colorectal cancer metastasis through upregulating ACLY and IGF1R
Source: Cell Death Dis. 2021 Jun 1;12(6):564. doi: 10.1038/s41419-021-03833-2 (PMC8169856; doi:10.1038/s41419-021-03833-2)
Supplement: Supplementary file 7 — Supplementary Table S5 [file 41419_2021_3833_MOESM7_ESM.docx]

Supplementary Table S5. Correlation between ACLY expression and clinicopathological characteristics of CRCs in two independent cohorts of human CRC tissues

|  |  | Cohort I (n=342) | |  |  | Cohort II (n=377) | |  |
| --- | --- | --- | --- | --- | --- | --- | --- | --- |
| Clinicopathological variables | | Tumor ACLY expression | | p Value |  | Tumor ACLY expression | | p Value |
|  |  | Negative  (n=178) | Positive (n=164) |  |  | Negative  (n=188) | Positive (n=189) |  |
| Age | | 65.51(11.58) | 66.98(10.97) | 0.228 |  | 67.91(11.46) | 67.09(11.75) | 0.491 |
| Sex | female | 74 | 76 | 0.385 |  | 81 | 88 | 0.535 |
|  | male | 104 | 88 |  |  | 107 | 101 |  |
| Tumor location | right colon | 83 | 67 | 0.509 |  | 72 | 88 | 0.180 |
|  | left colon | 75 | 74 |  |  | 91 | 74 |  |
|  | rectum | 20 | 23 |  |  | 25 | 27 |  |
| Tumor size | ＜5cm | 82 | 62 | 0.127 |  | 73 | 70 | 0.751 |
|  | ≥5cm | 96 | 102 |  |  | 115 | 119 |  |
| Tumor differentiation | well or moderate | 150 | 83 | <0.001 |  | 122 | 90 | 0.001 |
|  | poor | 28 | 81 |  |  | 66 | 99 |  |
| Tumor invasion | T1 | 5 | 2 | <0.001 |  | 11 | 4 | 0.211 |
|  | T2 | 23 | 7 |  |  | 11 | 13 |  |
|  | T3 | 125 | 99 |  |  | 132 | 129 |  |
|  | T4 | 25 | 56 |  |  | 34 | 43 |  |
| Lymph node metastasis | absent | 136 | 49 | <0.001 |  | 152 | 61 | <0.001 |
|  | present | 42 | 115 |  |  | 36 | 128 |  |
| Distant metastasis | absent | 164 | 113 | <0.001 |  | 169 | 136 | <0.001 |
|  | present | 14 | 51 |  |  | 19 | 53 |  |
| AJCC stage | Stage I | 28 | 7 | <0.001 |  | 14 | 5 | <0.001 |
|  | Stage II | 109 | 36 |  |  | 138 | 49 |  |
|  | Stage III | 27 | 70 |  |  | 18 | 83 |  |
|  | Stage IV | 14 | 51 |  |  | 18 | 52 |  |
