# Supplementary material for: IGF1-mediated HOXA13 overexpression promotes colorectal cancer metastasis through upregulating ACLY and IGF1R
Source: Cell Death Dis. 2021 Jun 1;12(6):564. doi: 10.1038/s41419-021-03833-2 (PMC8169856; doi:10.1038/s41419-021-03833-2)
Supplement: Supplementary file 8 — Supplementary Table S6 [file 41419_2021_3833_MOESM8_ESM.docx]

Supplementary Table S6. Correlation between IGF1R expression and clinicopathological characteristics of CRCs in two independent cohorts of human CRC tissues

|  |  | Cohort I (n=342) | |  |  | Cohort II (n=377) | |  |
| --- | --- | --- | --- | --- | --- | --- | --- | --- |
| Clinicopathological variables | | Tumor IGF1R expression | | p Value |  | Tumor IGF1R expression | | p Value |
|  |  | Negative  (n=153) | Positive (n=189) |  |  | Negative (n=170) | Positive (n=207) |  |
| Age | | 66.96(10.90) | 65.61(11.61) | 0.272 |  | 67.20(12.08) | 67.75(11.21) | 0.648 |
| Sex | female | 59 | 91 | 0.081 |  | 71 | 98 | 0.219 |
|  | male | 94 | 98 |  |  | 99 | 109 |  |
| Tumor location | right colon | 80 | 70 | 0.018 |  | 59 | 101 | 0.016 |
|  | left colon | 57 | 92 |  |  | 87 | 78 |  |
|  | rectum | 16 | 27 |  |  | 24 | 28 |  |
| Tumor size | ＜5cm | 62 | 82 | 0.660 |  | 63 | 80 | 0.831 |
|  | ≥5cm | 91 | 107 |  |  | 107 | 127 |  |
| Tumor differentiation | well or moderate | 136 | 97 | <0.001 |  | 106 | 106 | 0.037 |
|  | poor | 17 | 92 |  |  | 64 | 101 |  |
| Tumor invasion | T1 | 4 | 3 | <0.001 |  | 11 | 4 | <0.001 |
|  | T2 | 22 | 8 |  |  | 4 | 20 |  |
|  | T3 | 106 | 118 |  |  | 129 | 132 |  |
|  | T4 | 21 | 60 |  |  | 26 | 51 |  |
| Lymph node metastasis | absent | 124 | 61 | <0.001 |  | 139 | 74 | <0.001 |
|  | present | 29 | 128 |  |  | 31 | 133 |  |
| Distant metastasis | absent | 147 | 130 | <0.001 |  | 150 | 155 | 0.001 |
|  | present | 6 | 59 |  |  | 20 | 52 |  |
| AJCC stage | Stage I | 26 | 9 | <0.001 |  | 12 | 7 | <0.001 |
|  | Stage II | 98 | 47 |  |  | 125 | 62 |  |
|  | Stage III | 23 | 74 |  |  | 13 | 88 |  |
|  | Stage IV | 6 | 59 |  |  | 20 | 50 |  |
