# Supplementary material for: IGF1-mediated HOXA13 overexpression promotes colorectal cancer metastasis through upregulating ACLY and IGF1R
Source: Cell Death Dis. 2021 Jun 1;12(6):564. doi: 10.1038/s41419-021-03833-2 (PMC8169856; doi:10.1038/s41419-021-03833-2)
Supplement: Supplementary file 9 — Supplementary Table S7 [file 41419_2021_3833_MOESM9_ESM.docx]

Supplementary Table S7. Knockdown shRNA sequences used in this study

| TRC number | Sequence |
| --- | --- |
| HOXA13 |  |
| shRNA | CCGGTCGCGGACAAGTACATGGATACTCGAGTATCCATGTACTTGTCCGCGATTTTT |
| ACLY |  |
| TRCN0000078283 | CCGGGCCTCAAGATACTATACATTTCTCGAGAAATGTATAGTATCTTGAGGCTTTTTG |
| IGF1R |  |
| TRCN0000023492 | CCGGCAATGGTAACTTGAGTTACTACTCGAGTAGTAACTCAAGTTACCATTGTTTTT |
